# Efficacy and Safety of a Protein-Based SARS-CoV-2 Vaccine: A Randomized Clinical Trial

**DOI:** 10.1001/jamanetworkopen.2023.10302

**Published:** 2023-05-03

**Authors:** Ehsan Mostafavi, Sana Eybpoosh, Mohammad Karamouzian, Malahat Khalili, Saiedeh Haji-Maghsoudi, Mostafa Salehi-Vaziri, Ali Khamesipour, Tahmineh Jalali, Mehran Nakhaeizadeh, Hamid Sharifi, Yasaman Mansoori, Fariba Keramat, Samad Ghodrati, Mostafa Javanian, Delaram Doroud, Mir Davood Omrani, Hassan Asadi, Mohammad Hassan Pouriayevali, Roya Ghasemian, Hossein Farshidi, Morteza Pourahmad, Iman Ghasemzadeh, Leila Mounesan, Maryam Darvishian, Mohamad Reza Mirjalili, Maria Eugenia Toledo-Romani, Carmen Valenzuela-Silva, Vicente Verez-Bencomo, Mohammad Mehdi Gouya, Hamid Emadi-Koochak, Ali Akbar Haghdoost, Alireza Biglari

**Affiliations:** 1 Department of Epidemiology and Biostatistics, Research Centre for Emerging and Reemerging Infectious Diseases, Pasteur Institute of Iran, Tehran, Iran; 2 HIV/STI Surveillance Research Center, and WHO Collaborating Center for HIV Surveillance, Institute for Futures Studies in Health, Kerman University of Medical Sciences, Kerman, Iran; 3 School of Public Health, Brown University, Providence, Rhode Island; 4 Centre on Drug Policy Evaluation, St. Michael's Hospital, Toronto, Ontario, Canada; 5 The Michael G. DeGroote Institute for Pain Research and Care, McMaster University, Hamilton, Ontario, Canada; 6 Modeling in Health Research Center, Institute for Futures Studies in Health, Kerman University of Medical Sciences, Kerman, Iran; 7 Department of Biostatistics and Epidemiology, School of Public Health, Kerman University of Medical Sciences, Kerman, Iran; 8 COVID-19 National Reference Laboratory, Pasteur Institute of Iran, Tehran, Iran; 9 Center for Research and Training in Skin Diseases and Leprosy, Tehran University of Medical Sciences, Tehran, Iran; 10 Shiraz University of Medical Sciences, Shiraz, Iran; 11 Brucellosis Research Center, Hamadan University of Medical Sciences, Hamadan, Iran; 12 Internal Medicine Department, Zanjan University of Medical Sciences, Zanjan, Iran; 13 Zanjan Metabolic Diseases Research Center, School of Medicine, Zanjan University of Medical Sciences, Zanjan, Iran; 14 Infectious Diseases and Tropical Medicine Research Center, Health Research Institute, Babol University of Medical Sciences, Babol, Iran; 15 Quality Control Department, Production and Research Complex, Pasteur Institute of Iran, Tehran, Iran; 16 Department of Genetics, School of Medicine, Urogenital Stem Cell Research Center, Shahid Beheshti University of Medical Sciences, Tehran, Iran; 17 Pasteur Institute of Iran, Tehran, Iran; 18 Department of Health Information Management, School of Health Management and Information Sciences, Iran University of Medical Sciences, Tehran, Iran; 19 Department of Infectious Diseases, Antimicrobial Resistance Research Center, Mazandaran University of Medical Sciences, Sari, Iran; 20 Cardiovascular Research Center, Hormozgan University of Medical Sciences, Bandar Abbas, Iran; 21 Department of Infectious Diseases, Infectious Diseases and Tropical Medicine Research Center, Isfahan University of Medical Sciences, Isfahan, Iran; 22 Research Center of Tropical and Infectious Diseases, Kerman University of Medical Sciences, Kerman, Iran; 23 Cancer Control Research, BC Cancer Research Centre, Vancouver, British Columbia, Canada; 24 Shahid Sadoughi University of Medical Sciences, Yazd, Iran; 25 Pedro Kourí Tropical Medicine Institute, Havana, Cuba; 26 Cybernetics, Mathematics and Physics Institute, Havana, Cuba; 27 Finlay Vaccine Institute, Havana; 28 Centre for Communicable Disease Control, Ministry of Health and Medical Education, Tehran, Iran; 29 Department of Infectious Disease and Tropical Medicine, School of Medicine, Iran University of Medical Sciences, Tehran, Iran; 30 Department of Infectious Disease, School of Medicine, Imam Khomeini Hospital, Tehran University of Medical Sciences, Tehran, Iran; 31 School of Medicine, Tehran University of Medical Sciences, Tehran, Iran

## Abstract

**Importance:**

The protein-based SARS-CoV-2 vaccines FINLAY-FR-2 (Soberana 02) and FINLAY-FR-1A (Soberana Plus) showed good safety and immunogenicity in phase 1 and 2 trials, but the clinical efficacy of the vaccine remains unknown.

**Objective:**

To evaluate the efficacy and safety of a 2-dose regimen of FINLAY-FR-2 (cohort 1) and a 3-dose regimen of FINLAY-FR-2 with FINLAY-FR-1A (cohort 2) in Iranian adults.

**Design, Setting, and Participants:**

A multicenter, randomized, double-blind, placebo-controlled, phase 3 trial was conducted at 6 cities in cohort 1 and 2 cities in cohort 2. Participants included individuals aged 18 to 80 years without uncontrolled comorbidities, coagulation disorders, pregnancy or breastfeeding, recent immunoglobulin or immunosuppressive therapy, and clinical presentation or laboratory-confirmed COVID-19 on enrollment. The study was conducted from April 26 to September 25, 2021.

**Interventions:**

In cohort 1, 2 doses of FINLAY-FR-2 (n = 13 857) or placebo (n = 3462) were administered 28 days apart. In cohort 2, 2 doses of FINLAY-FR-2 plus 1 dose of FINLAY-FR-1A (n = 4340) or 3 placebo doses (n = 1081) were administered 28 days apart. Vaccinations were administered via intramuscular injection.

**Main Outcomes and Measures:**

The primary outcome was polymerase chain reaction–confirmed symptomatic COVID-19 infection at least 14 days after vaccination completion. Other outcomes were adverse events and severe COVID-19. Intention-to-treat analysis was performed.

**Results:**

In cohort 1 a total 17 319 individuals received 2 doses and in cohort 2 5521 received 3 doses of the vaccine or placebo. Cohort 1 comprised 60.1% men in the vaccine group and 59.1% men in the placebo group; cohort 2 included 59.8% men in the vaccine group and 59.9% in the placebo group. The mean (SD) age was 39.3 (11.9) years in cohort 1 and 39.7 (12.0) years in cohort 2, with no significant difference between the vaccine and placebo groups. The median follow-up time in cohort 1 was 100 (IQR, 96-106) days and, in cohort 2, 142 (137-148) days. In cohort 1, 461 (3.2%) cases of COVID-19 occurred in the vaccine group and 221 (6.1%) in the placebo group (vaccine efficacy: 49.7%; 95% CI, 40.8%-57.3%) vs 75 (1.6%) and 51 (4.3%) in cohort 2 (vaccine efficacy: 64.9%; 95% CI, 49.7%-59.5%). The incidence of serious adverse events was lower than 0.1%, with no vaccine-related deaths.

**Conclusions and Relevance:**

In this multicenter, randomized, double-blind, placebo-controlled, phase 3 trial of the efficacy and safety of FINLAY-FR-2 and FINLAY-FR-1A, 2 doses of FINLAY-FR-2 plus the third dose of FINLAY-FR-1A showed acceptable vaccine efficacy against symptomatic COVID-19 as well as COVID-19–related severe infections. Vaccination was generally safe and well tolerated. Therefore, Soberana may have utility as an option for mass vaccination of the population, especially in resource-limited settings, because of its storage condition and affordable price.

**Trial Registration:**

isrctn.org Identifier: IRCT20210303050558N1

## Key Points

**Question:**

Do the protein-based SARS-CoV-2 vaccines (FINLAY-FR-2 and FINLAY-FR-1A) provide safe and effective protection against SARS-CoV-2?

**Findings:**

In this randomized clinical trial including 23 959 individuals, vaccine efficacy for prevention of polymerase chain reaction (PCR)–positive symptomatic COVID-19 was 49.7%; severe disease, 76.8%; and hospitalization, 77.7% after the 2-dose regimen of FINLAY-FR-2, which improved to 64.9% for prevention of PCR-positive symptomatic COVID-19, 96.6% for prevention of severe disease, and 96.6% for prevention of hospitalization in recipients of a 2-dose regimen of FINLAY-FR-2 and a third dose of FINLAY-FR-1A. The incidence of serious adverse events was lower than 0.1%.

**Meaning:**

The findings of this trial suggest that FINLAY-FR-2, in combination with a third dose of FINLAY-FR-1A, is a safe vaccine inducing a potent immune response against COVID-19.

## Teaser

This randomized clinical trial evaluates the efficacy and safety of the FINLAY-FR-2 and FINLAY-FR-1A COVID-19 vaccines in the Iranian population.

## Introduction

Vaccines have been considered one of the most important interventions for curbing the COVID-19 pandemic. COVID-19 vaccine studies have been conducted based on different approaches including the first-generation platforms (eg, attenuated and inactivated virus vaccines), the second-generation vaccines, such as replicating vector vaccines and protein-based vaccines (eg, subunit and viral vectors), and the third-generation vaccines, such as nanoparticle and genetic vaccines (eg, DNA/RNA vaccines).^1^ Several COVID-19 vaccines are developed from the SARS-CoV-2 spike glycoprotein receptor-binding domain (RBD) to prevent the virus attachment to human cell angiotensin-converting enzyme 2 receptors.

FINLAY-FR-2 (Soberana 02) is a protein subunit vaccine conjugated to the tetanus toxoid carrier protein and has shown potential in preclinical studies^2,3^ and phase 1, 2, and 3 trials.^4,5,6,7^ FINLAY-FR-1A (Soberana Plus), which is an RBD dimer without conjugation, has enhanced neutralization response in individuals recovering from COVID-19^8^ and, when used as the third dose to FINLAY-FR-2, induced neutralizing anti-RBD immunoglobulin G (IgG) antibodies (eMethods in Supplement 2).^4^ FINLAY-FR-2 and FINLAY-FR-1A have received permission for emergency use in Belarus, Cuba, Iran, Mexico, Nicaragua, and Venezuela. FINLAY-FR-2 and FINLAY-FR-1A vaccines are developed and produced at the Finlay Vaccine Institute of Cuba and branded as Pastocovac and Pastocovac Plus, and are manufactured by the Pasteur Institute of Iran after a successful technology transfer.

On approval of the first sets of COVID-19 vaccines, governments all over the world rushed to place orders for supplies, based on the assumed effectiveness and relevance of the vaccine for their populations. While the recent launch of several safe and efficient COVID-19 vaccines has offered hope to tens of millions of people, vaccine supply is limited in low-income countries where the governments face supply challenges posed by vaccine cost as well as storage and transportation conditions.^9^ The potential advantage of RBD-based vaccines is their rapid and affordable production, ease of scalability, and stability at 2 to 8 °C,^10^ while the presence of multiple T- and B-cell epitopes of tetanus toxoid carriers, as part of a conjugate vaccine, potentiates cellular immune response.^2^

We report a double-blind, randomized, placebo-controlled phase 3 vaccine trial to assess the efficacy, safety, and immunogenicity of FINLAY-FR-2 with the third dose of FINLAY-FR-1A, 50 μg, in Iran, as a country that has not reported on many trials.

## Methods

### Study Design and Participants

A multicenter, randomized, double-blind, parallel-group, placebo-controlled phase 3 clinical trial was conducted to evaluate the efficacy, safety, and immunogenicity of a 2-dose regimen of conjugated protein-based FINLAY-FR-2, 25 μg, in one cohort (cohort 1) and a 2-dose regimen of FINLAY-FR-2 with a third dose of FINLAY-FR-1A, 50 μg, in a second cohort (cohort 2) in Iran. The study was conducted from April 26 to September 25, 2021. The trial protocol was reviewed and approved by Iran’s Food and Drug Administration and the National Committee for Ethics in Biomedical Research. The study followed the Consolidated Standards of Reporting Trials (CONSORT) reporting guideline.^11^

Participants were recruited from 8 geographically dispersed cities between April 26 and May 23, 2021 (eFigure 1 in Supplement 2) on signing written informed consent. Participants received financial compensation. Cohort 1 included 17 319 volunteers (resided in 6 cities), and cohort 2 included 5521 volunteers (lived in 2 cities) (eAppendix in Supplement 2).

Volunteers aged 18 to 80 years without a current clinical presentation or laboratory-confirmed COVID-19 or history of COVID-19 vaccination, congenital or uncontrolled type 2 diabetes, chronic kidney disease, hypertension, and chronic liver disease could be included. The details of eligibility criteria are provided in the trial protocol (Supplement 1).

During the study, Iran initiated an immediate COVID-19 vaccination program, prioritizing older adults (≥65 years). To comply with the ethical principles,^12^ and following the recommendation of the National Committee for Ethics in Biomedical Research, blinding for participants aged 65 years or older was discontinued (aged ≥70 years: May 25, 2021; aged 65-70 years, July 13, 2021). These people were given priority in the national vaccination program to receive an approved vaccine and were excluded from analysis.

### Randomization

Randomization was conducted using a stratified balanced block randomization approach (block size: 25, stratum variable: city) on day 0, at a 4:1 ratio. Participants, study staff, and investigators were blinded for group allocation. Allocations were concealed through the central assignment. The vaccine and placebo vials were identical and indistinguishable in appearance.

### Procedures

FINLAY-FR-2 was administered via intramuscular injection, 28 days apart. FINLAY-FR-1A was used as a third dose in cohort 2 on day 56. Vaccine composition is presented in the eMethods in Supplement 2.

QuantiVac ELISA (enzyme-linked immunosorbent assay IgG) kit (Euroimmun, Germany) was used to determine the titration of anti-S1 IgG (including RBD) antibodies. To evaluate vaccine immunogenicity, a representative sample of participants (30% of the total) was selected from randomly selected cities on days 0 and 56 in cohort 1 (n = 5905) and days 0 and 84 in cohort 2 (n = 1975). Microneutralization assay^13^ was also performed in a proportion of anti-S1 IgG ELISA-positive participants on days 56 (cohort 1; n = 54) and 84 (cohort 2; n = 58) using SARS-CoV-2 (hCoV-19/Iran/AK-SARS-A7/2020). Interferon-γ was assessed on day 56 using the SARS-CoV-2 interferon gamma release assay (Euroimmun). To rule out the possibility of natural infection interference with the interferon-γ release assay and neutralization tests, samples were collected from participants who had IgG antibodies on day 0 or those who tested positive in real-time polymerase chain reaction (RT-PCR) testing until the immunogenicity sampling times were excluded.

Participants with suspected COVID-19 (based on symptoms) were tested using a 1-step RT-PCR kit (Pishtaz Teb Diagnostics), with the findings confirmed with the 2019-nCoV Nucleic Acid Diagnostic Kit (Sansure Biotech). Participants with RT-PCR–positive testing with an N gene cycle threshold value less than 25 underwent an S gene sequence analysis.^14^ SARS-CoV-2 variant determination was conducted using the Nextclade application.^15^ All sequences were submitted to the Global Initiative on Sharing Avian Influenza Data (eMethods in Supplement 2).

### Outcomes

Consistent with other vaccine trials, the primary outcome was symptomatic COVID-19, confirmed by RT-PCR 14 days after the second and third doses. Secondary outcomes were severe COVID-19, COVID-19–related death, adverse events (AEs), and humoral and cellular immune responses (eMethods in Supplement 2).

Participants recorded solicited AEs (ie, set of symptoms or events that study participants are explicitly requested to document) using an online platform within 3 days after receiving each injection. Unsolicited AEs were followed up 28 days after each dose. Within 28 days after the injection of each dose, participants were followed up for any type of AEs (mildly or moderately severe, AEs with medically attended visits, or life-threatening AEs) on day 3 and then weekly through telephone calls. From day 28 until the end of the follow-up period, telephone follow-ups were performed every 2 weeks. Also, participants were supported with a continuously operating call center and medical visits. Medically attended AEs and serious AEs were monitored 6 months after the first dose in cohort 1 and 7 months after the first dose in cohort 2.

### Statistical Analysis

The sample size was calculated to be approximately 18 000 participants in cohort 1 (vaccine: 14 400, placebo: 3600) and 6000 in cohort 2 (vaccine: 4800, placebo: 1200) (eMethods in Supplement 2). Intention-to-treat analysis was performed. Vaccine efficacy was defined as the reduction in the hazard ratio for the symptomatic COVID-19 (vaccine vs placebo), using a Cox proportional hazards regression model stratified by city. It was calculated as a 1 – *exp(β)* of vaccine group vs the placebo group. The geometric mean titer ratio was used to calculate 95% CIs with the nonparametric percentile bootstrap method with 1000 repetitions. A bootstrap *t* test was used to compare log-transformed titers between the vaccine and placebo groups.

Seroconversion was defined as a 4-fold increase in anti–SARS-CoV-2 S1 IgG titer compared with the baseline. Adverse events were compared between the vaccine and placebo groups using the χ^2^ or Fisher exact tests. Risk ratios for any AEs are reported. For safety analysis, AEs of cohort 1 and cohort 2 in the first and second doses were combined. All statistical analyses were conducted in R, version 4.1.0 software (R Foundation for Statistical Computing). The testing for significance was 2-sided, with a threshold of .05. The comparison between the vaccine and placebo groups and the analysis of AEs were unpaired, while the analysis of the difference in antibody titer between day 0 and subsequent days was paired. Further details on statistical analysis are provided in the eMethods in Supplement 2.

## Results

### Participants

**Figure 1.**
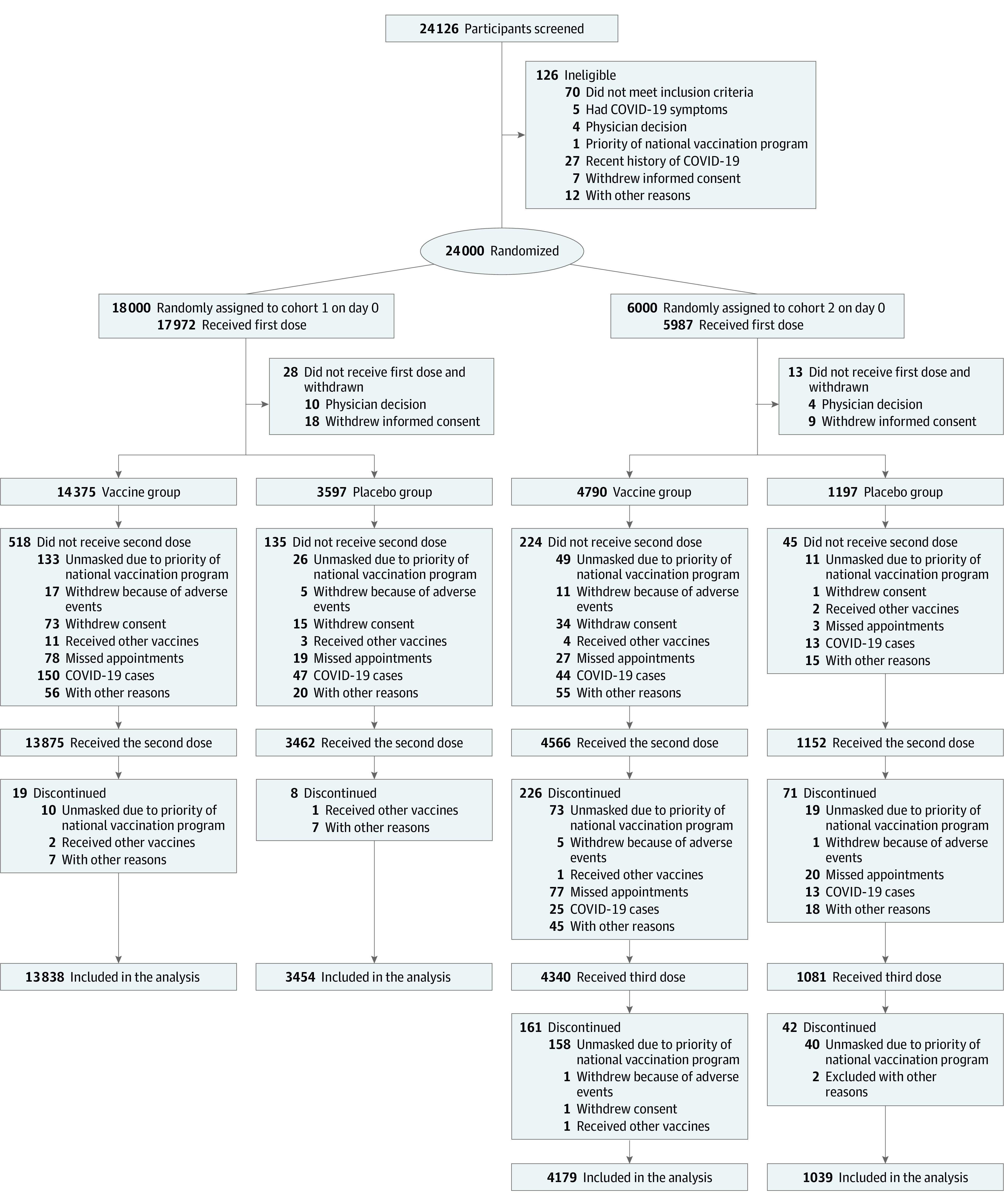
Trial Profile Reasons for unmet inclusion criteria in screened participants are reported in eTable 10 in Supplement 2.

Between April 26 and May 23, 2021, of 24 126 volunteers screened, 18 000 were randomized to placebo or vaccine in cohort 1 and 6000 were randomized to placebo or vaccine in cohort 2. A total of 23 959 participants (cohort 1 [n = 17 972] and cohort 2 [n = 5987]) were randomly assigned to receive the vaccine (n = 19 165) or placebo (n = 4794) (Figure 1). Overall, the mean (SD) age was 39.3 (11.9) years in cohort 1 and 39.7 (12.0) years in cohort 2, with no significant difference between the vaccine and placebo groups. In cohort 1, participants’ mean (SD) age was 39.4 (11.9) years in the vaccine group (n = 14 375; 8637 [60.1%] men, 5738 [39.9%] women) and 39.1 (11.7) years in the placebo group (n = 3597; 2127 [59.1%] men, 1470 [40.9%] women). In cohort 2, the mean (SD) age was 39.6 (12.1) years in the vaccine group (2866 [59.8%] men, 1924 [40.2%] women) and 39.9 (11.7) years in the placebo group (717 [59.9%] men, 480 [40.1%] women). At least 1 underlying disease was reported in 5208 (29.0%) participants in cohort 1 and 1798 (30.0%) in cohort 2.

Of participants who underwent a serologic test on day 0 (cohort 1, 5905; cohort 2, 1975), 35.7% of those in the cohort 1 vaccine group and 33.7% of those in the cohort 2 vaccine group, as well as 36.7% of those in the cohort 1 placebo group and 29.7% in the cohort 2 placebo group, had anti–SARS-CoV-2 S1 IgG (Table 1).

**Table 1.** Baseline Characteristics of the Participants Who Received at Least 1 Dose of the Assigned Treatment

Characteristic	No. (%)			
	Cohort 1		Cohort 2	
	Vaccine (n = 14 375)	Placebo (n = 3597)	Vaccine (n = 4790)	Placebo (n = 1197)
Age, y				
Mean (SD)	39.4 (11.9)	39.1 (11.7)	39.6 (12.1)	39.9 (11.7)
≤65	14 031 (97.6)	3511 (97.6)	4676 (97.6)	1169 (97.7)
Sex				
Men	8637 (60.1)	2127 (59.1)	2866 (59.8)	717 (59.9)
Women	5738 (39.9)	1470 (40.9)	1924 (40.2)	480 (40.1)
Educational level				
Illiterate	196 (1.4)	42 (1.2)	73 (1.5)	17 (1.4)
High school	1370 (9.5)	355 (9.9)	556 (11.6)	139 (11.6)
Diploma or postdiploma	4942 (34.4)	1240 (34.5)	1471 (30.7)	356 (29.7)
Bachelor’s degree	4825 (33.6)	1225 (34.1)	1644 (34.3)	412 (34.4)
≥Master’s degree	3042 (21.2)	735 (20.4)	1046 (21.8)	273 (22.8)
Underlying diseases^a^				
Yes	4253 (29.6)	973 (27.1)	1446 (30.2)	352 (29.4)
BMI				
Mean (SD)	26.6 (4.6)	26.5 (4.6)	27.2 (4.5)	27.3 (4.6)
<25	5410 (37.6)	1407 (39.1)	1540 (32.2)	373 (31.2)
25-30	5887 (41.0)	1457 (40.5)	2113 (44.1)	533 (44.5)
>30	3078 (21.4)	733 (20.4)	1137 (23.7)	291 (24.3)
Anti–SARS-CoV-2 S1 IgG on day 0^b^				
Positive	1684 (35.7)	436 (36.7)	538 (33.7)	113 (29.7)
Negative	3034 (64.3)	751 (63.3)	1057 (66.3)	267 (70.3)

Abbreviations: BMI, body mass index (calculated as weight in kilograms divided by height in meters squared); IgG, immunoglobulin G.

^a^ Underlying diseases: obesity (BMI >30), controlled hypertension, chronic kidney disease, chronic liver disease, type 2 diabetes, chronic obstructive pulmonary disease), controlled asthma, history of any malignancy or cancer, and ischemic heart disease. The details are provided in the protocol (Supplement 1).

^b^ IgG antibodies against the S1 subunit (S1 IgG).

### Immunologic Response

In the vaccine group, the seroconversion rates of anti–SARS-CoV-2 S1 IgG increased 4 weeks after the second (81.1% [95% CI, 79.7%-82.2%]) and third (92.9% [95% CI, 91.4%-94.2%]) doses (*P* < .001) (eTable 4 in Supplement 2). For neutralizing antibodies, the rates were 69.8% (95% CI, 53.8%-82.8%) after the second dose and 100% after the third dose (eFigure 3 and eMethods in Supplement 2).

A specific IFN-γ response to SARS-CoV-2 S1 was analyzed in 363 participants 4 weeks after the second dose (295 from the vaccine group and 68 from the placebo group). The geometric mean titer ratio of IFN-γ between the vaccine and placebo groups was 5.3 (95% CI, 3.1-9.2; *P* = .001).

### Efficacy

**Figure 2.**
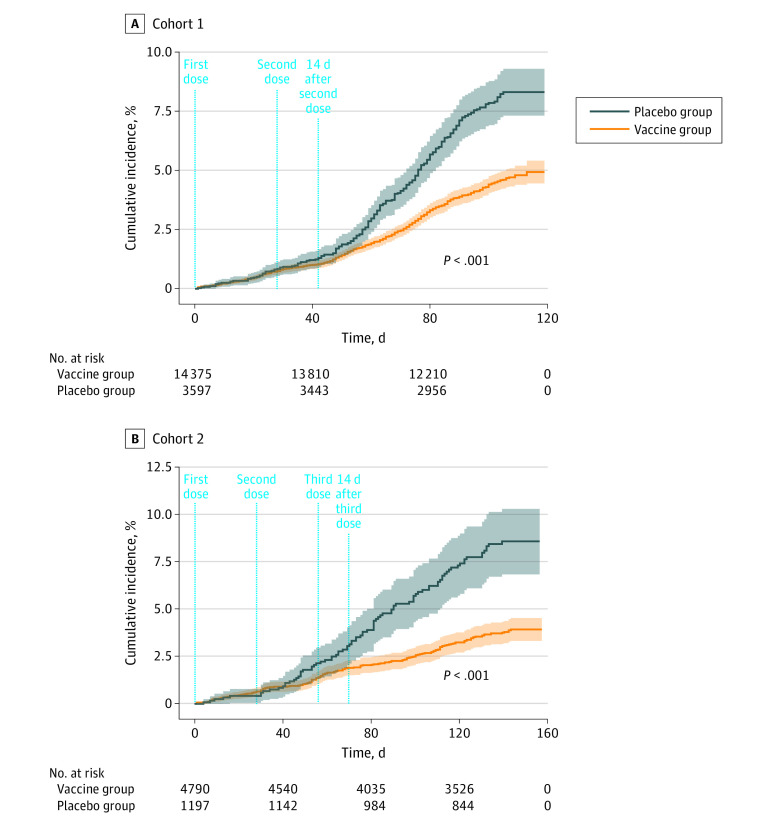
Cumulative Incidence of COVID-19 Incident Cases (1 – Kaplan-Meier Estimate) in the Primary Analysis Following the First Dose of Vaccine or Placebo in the 2-Dose and 3-Dose Regimens

The median follow-up time for cohort 1 was 100 days (IQR, 96-106 days), and for cohort 2, 142 days (IQR, 137-148 days). In cohort 1, 461 (3.2%) symptomatic cases in the vaccine group and 221 (6.1%) in the placebo group were observed, which yielded a vaccine efficacy (VE) of 49.7% (95% CI, 40.8%-57.3%). In cohort 2, 75 (1.6%) symptomatic cases occurred in the vaccine group and 51 (4.3%) in the placebo group, corresponding to a VE of 64.9% (95% CI, 49.7%-59.5%) (Figure 2; Table 2; eFigure 2 in Supplement 2). Among participants who tested negative for anti–SARS-CoV-2 S1 IgG on day 0, VE was relatively comparable to the VE in the total sample (cohort 1: 54.7%; 95% CI, 35.0%-68.5% and cohort 2: 64.1%; 95% CI, 34.0%-80.5%) (eResults in Supplement 2). Vaccine efficacy for the prevention of severe cases in cohort 1 was 76.8% (95% CI, 61.7%-86.0%) and for prevention of COVID-19–related hospitalization was 77.7% (95% CI, 60.9%-87.4%). Vaccine efficacy for the prevention of severe cases after the third dose was 96.6% (95% CI, 72.2-99.6) and for prevention of hospitalization was 96.6% (95% 5 CI, 72.2%-99.6%) (Table 2). During the follow-up period, only 1 COVID-19–related death was detected in the placebo group; therefore, estimation of the VE for the prevention of COVID-19–related death was not feasible. The VE estimates based on logistic regression model are provided in eTable 2 and eTable 3 in Supplement 2. The VEs in different subgroups on the prevention of various outcomes based on age, sex, and underlying diseases are presented in eTable 1 of Supplement 2. The number of participants needed to vaccinate in cohort 1 was 32.3 and in cohort 2 was 29.4 (eTable 9 in Supplement 2).

**Table 2.** Vaccine Efficacy Against SARS-CoV-2, 14 Days After the Second and Third Doses in Cohort 1 and Cohort 2 Among the Vaccine and Placebo Groups

Condition^a^	Total cases, No.	No./total No. (%)		Vaccine efficacy, % (95% CI)^b^
		Placebo	Vaccine	
**Cohort 1: 14 d after the second dose**				
Confirmed symptomatic COVID-19	682	221/3600 (6.1)	461/14 400 (3.2)	49.7 (40.8-57.3)
Severe cases	51	25/3600 (0.7)	26/14 400 (0.2)	76.8 (61.7-86.0)
Hospitalization	48	25/3600 (0.7)	23/14 400 (0.2)	77.7 (60.9-87.4)
**Cohort 2: 14 d after the third dose**				
Confirmed symptomatic COVID-19	126	51/1200 (4.3)	75/4800 (1.6)	64.9 (49.7-59.5)
Severe cases	8	7/1200 (0.6)	1/4800 (0.0)	96.6 (72.2-99.6)
Hospitalization	8	7/1200 (0.6)	1/4800 (0.0)	96.6 (72.2-99.6)

^a^ Confirmed symptomatic COVID-19 indicates the primary outcome, and severe cases as the secondary outcome; only 1 COVID-19–related death was observed in the placebo group during the follow-up period in cohort 1.

^b^ Vaccine efficacy was defined as a 1 – *exp(β)* of vaccine group vs the placebo group.

The spike gene sequencing analysis of 419 participants with RT-PCR–positive testing revealed that the Alpha variant was dominant before June 10, 2021 (when the second dose was administered). However, 1 month after the second dose, Delta became the dominant variant as of August 2021 when 100% of individuals with positive RT-PCR testing were infected with the Delta variant (eFigure 4 in Supplement 2).

Consistent with other vaccine trials,^16,17^ the infections occurring during 14 days after the second and third doses were not included in the efficacy analyses. In cohort 1, 2 (placebo group) and 3 (vaccine group) participants had infections during the 14 days following the second dose. In cohort 2, the number of participants who experienced infections in this period was 2 (placebo group) and 1 (vaccine group).

### Safety

**Figure 3.**
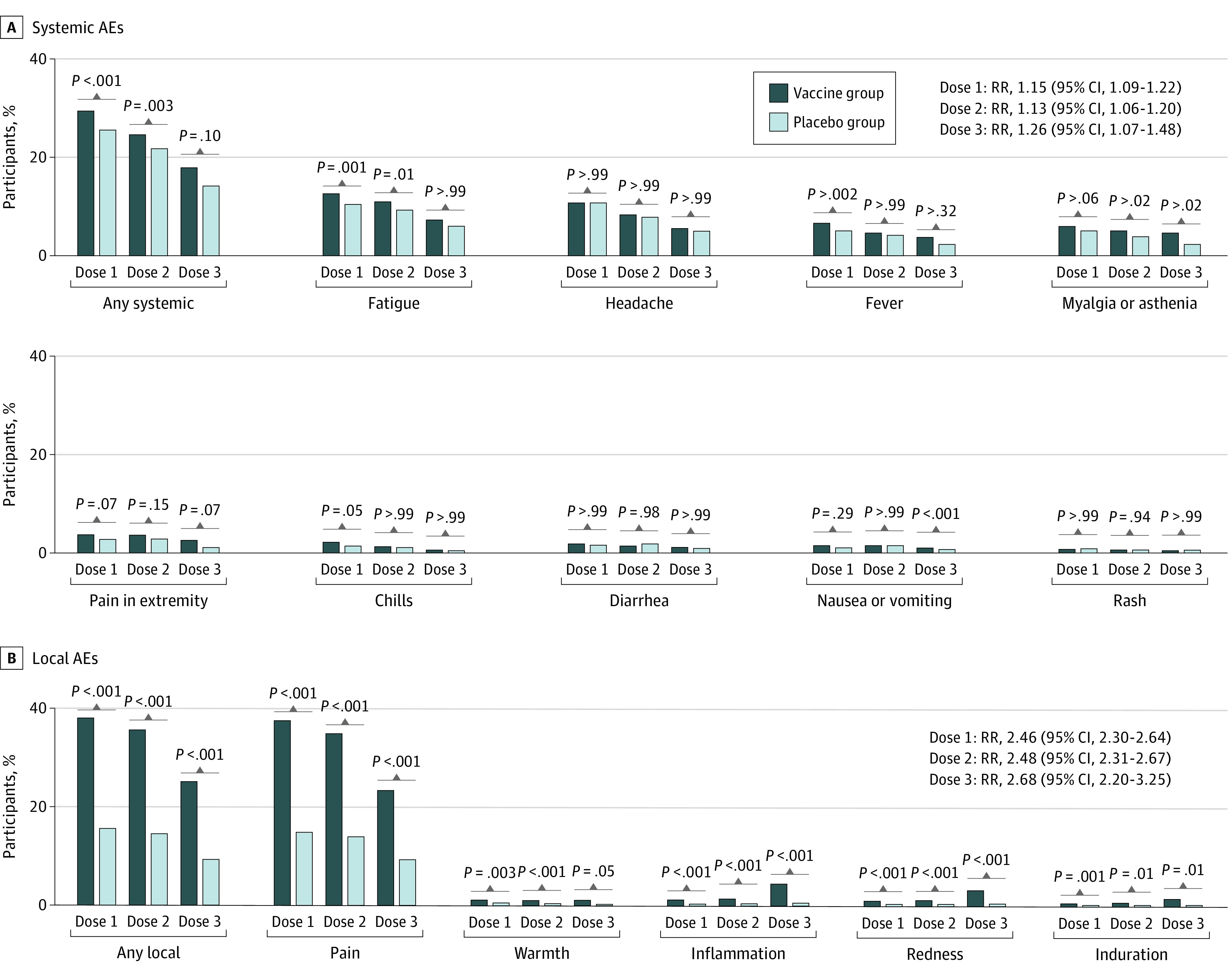
Local and Systemic Adverse Events After Injection of the First, Second, and Third Doses Among Participants Who Received Vaccine or Placebo AEs indicates adverse events; RR, risk ratio.

Solicited AEs at the injection site were reported more frequently in the vaccine group than the placebo group after the first dose (7269 [38.2%] vs 737 [15.5%]), second dose (6470 [35.7%] vs 649 [14.4%]), and third dose (1042 [25.0%] vs 98 [9.3%]) (Figure 3; eTable 5 in Supplement 2). Local pain at the injection site was the most common AE, reported in 35.1% of the participants in the vaccine group and 13.9% in the placebo group (Figure 3; eTable 6 in Supplement 2).

Systemic solicited AEs were more common in the vaccine group than in the placebo group after the first dose (5585 [29.3%] vs 1211 [25.5%]), second dose (4427 [24.4%]) vs 977 [21.7%]), and third dose (739 [17.7%] vs 148 [14.1%]). Fatigue was the most common systemic AE in both the vaccine (dose 1: 2380 [12.5%], dose 2: 1971 [10.9%], and dose 3: 299 [7.2%]) and placebo (dose 1: 492 [10.4%], dose 2: 411 [9.1%], and dose 3: 62 [5.9%]) groups (Figure 3; eTable 6 in Supplement 2).

Unsolicited AEs within 3 days after the injection were comparable among participants in the vaccine and placebo groups after the first (1696 [8.9%] vs 458 [9.6%]) and second (1324 [7.3%] vs 334 [7.4%]) doses. However, in cohort 2, after the injection of FINLAY-FR-1A, there was a significant difference between the vaccine and placebo groups (330 [7.9%] vs 55 [5.2%]) (*P* = .003) (eTable 5 in Supplement 2). Moreover, 37 participants had medically attended AEs (31 [0.2%] in the vaccine group and 6 [0.1%] in the placebo group) (eTable 6 in Supplement 2), 4 of which were associated with the vaccine (2 injection site reactions, 1 generalized itching, and 1 abdominal pain and fecal impaction) and 1 with the placebo (lower limb paresthesia and vomiting).

Overall, 23 serious AEs (19 [0.1%] in the vaccine group and 4 [<0.1%] in the placebo group) were reported, none of which were associated with the vaccine (eTable 7 in Supplement 2). Four (<0.1%) deaths were reported in the vaccine group (1 sudden cardiac death, 1 brain death after ventricular arrhythmias and cardiac arrest, 1 death of unknown cause, and 1 road accident) and 2 (<0.1%) in the placebo group (1 from ischemic heart disease and 1 from cardiorespiratory arrest with underlying stroke and hypertension), none of which were related to the vaccine (eTable 8 in Supplement 2). Moreover, 2 COVID-19–related deaths were reported in the placebo group of cohort 1, 1 of which occurred during the follow-up period for VE (57 days after the second dose), and 1 happened 22 days after unblinding (98 days after the second dose). One COVID-19–related death occurred in the vaccine group of cohort 1 10 days after the end of the follow-up period for VE and unblinding (78 days after the second dose).

## Discussion

Our analysis showed that the VE of 2 doses of FINLAY-FR-2 (cohort 1) in preventing symptomatic infections was 49.7% of the participants, severe cases in 76.8%, and COVID-19–related hospitalizations in 77.7%. Vaccine efficacy was higher in recipients of the third dose (cohort 2), at 64.9%, for preventing symptomatic infections and 96.6% for preventing severe cases and hospitalizations.

Subunit protein vaccines are one of the most viable options for resource-limited settings due to their possible storage in refrigerators and easier distribution.^18,19,20^ These vaccines have shown potential utility in preclinical animal studies and controlled trials.^2,8,21,22,23,24,25^ The preclinical results with the vaccine highlighted the advantages of immunization with the recombinant RBD–tetanus toxoid conjugates and showed that it could trigger a robust immune response.^2,3^

Findings of a phase 3 randomized clinical trial on FINLAY-FR-2 in 44 031 participants in Cuba reported VE against PCR-confirmed COVID-19 cases to be 69.7% (95% CI, 56.5%-78.9%) for the 2-dose and 92.0% (95% CI, 80.4%-96.7%) for the 3-dose regimen.^5^ Our trial’s VE against symptomatic disease has been lower, particularly for the 3-dose regimen. The observed differences between VE estimates against symptomatic disease could be partly explained by the differences in the force of infection, presence of comorbidities in the studied populations, and circulating SARS-CoV-2 variants before and during the trials in both countries. Although the case definitions of symptomatic disease in both trials have been equivalent, the cultural representation of the various signs and symptoms may vary between the 2 settings, resulting in patients presenting with a different clinical picture being selected for PCR confirmation.

The VE assessed in this study should be interpreted in the context of the dominance of the immune-evasive Delta variant during the follow-up period.^26,27^ A study in England suggested that the effectiveness of the BNT162b2 vaccine decreased from 93.7% (95% CI, 91.6%-95.3%) among people with the Alpha variant to 88.0% (95% CI, 85.3%-90.1%) in the Delta variant.^28^ Studies have highlighted lowered VE of BNT162b2 vaccine against new infections in Qatar (64.2% [95% CI, 38.1%-80.1%]),^29^ Israel (39% [95% CI, 9.0%-59.0%]),^30^ and the US (42% [95% CI, 13.0%-62.0%]).^31^ The VE of mRNA-1273 vaccine against the Delta variant has also shown a modest reduction (2.1-fold) in neutralizing titers.^26^ Moreover, the VE of ChAdOx1 nCoV-19 vaccine appears to be reduced against the Delta (67.0% [95% CI, 61.3%-71.8%] vs Alpha (74.5% [95% CI, 68.4%-79.4%]) variant.^28^ Although our understanding of the adverse effects of vaccine-escaping variants on the existing VEs continues to increase, the reduced acquired immunity could partly explain the decreased VE estimates over time.

This trial benefited from a large sample size and a cohort of geographically diverse volunteers across the country. FINLAY-FR-2 is one of the only vaccines that noted the need for a third dose from its early design phases and could serve as an accessible, scalable, and affordable option for resource-limited settings that have faced challenges in accessing an equitable vaccine supply.

In this study, we only excluded individuals with current COVID-19 and included others regardless of their COVID-19 history. This was recommended by the Iran Food and Drug Administration committee based on several considerations. The study started about 15 months after the onset of the epidemic in Iran. Within this period, a substantial proportion of the population contracted SARS-CoV-2 infection. However, COVID-19 history could not be verified through PCR test records, as many individuals with actual positive infection did not undergo testing at the early stages of the epidemic. Even if we had used negative antibody titers at baseline as an inclusion criterion, we would have erroneously included many individuals with a history of COVID-19 who had been infected at the early stages of the pandemic and had undetectable antibody titers due to the waning of their antibodies over time. The effect of serostatus on VE still needs further investigation. In a well-powered study conducted at the same time as our trial,^32^ large proportions of the participants were seropositive at baseline (44%), and the VE for the complete analysis set (66.7%) and seronegative population (67.2%) were similar. These data are comparable with our results, which showed that the VE in the naive population was similar to that of the total population.

### Limitations

We acknowledge the limitations of this study. First, up to 30% of volunteers with controlled underlying diseases were selected. This may have impacted the overall VE in this study. Second, we could not run serologic tests for all our participants at baseline due to our limited resources in Iran. Third, given that the older population had undergone mass vaccination during the study period, this age group underwent premature unblinding to receive approved vaccines, a procedure that could lead to selection bias due to the nonrandom exclusion of this group postrandomization; it was an ethical decision and was based on good clinical practice standards. Also, the magnitude of this bias was not considerable (approximately 2.5% of the total sample).

## Conclusions

In this multicenter, randomized, double-blind, placebo-controlled, phase 3 clinical trial, the study findings indicate that the FINLAY-FR-2 vaccine, in addition to the third dose of FINLAY-FR-1A, has acceptable VE against symptomatic cases and high efficacy against severe cases of COVID-19 and COVID-19–related hospitalizations. FINLAY-FR-2 and FINLAY-FR-1A also appeared to be well tolerated and have a favorable safety profile.

## Notes

Supplement 1.
Trial Protocol

Supplement 2. **eFigure 1.** Study Sites of the SOBERANA trial in Iran **eAppendix.** List of Participating Sites and Address of Different Units Within Each City **eMethods.** Detailed Methods **eFigure 2.** Schoenfeld Residual Plots and Estimated Log-Log Survival Curves for Assessing the Proportional Hazard Assumption **eTable 1.** Vaccine Efficacy Against SARS-CoV-2 14 Days in Different Subgroups Based on Stratified Cox Regression **eTable 2.** Vaccine Efficacy Against SARS-CoV-2 Using the Logistic Model **eTable 3.** Vaccine Efficacy Against SARS-CoV-2 in Different Subgroups Based on Logistic Regression **eResults.** Detailed Results **eFigure 3.** Humoral Immune Response (Anti SARS-CoV-2 S1 IgG and Neutralizing Antibodies Titers) in the Vaccine and Placebo Groups **eTable 4.** Seroconversion Rate in Different Subgroups After the First, Second, and Third Doses **eTable 5.** Adverse Events (AEs) From Day 0 to Day 28 **eTable 6.** Local and Systemic Adverse Events After Injection of the First, Second and Third Doses **eTable 7.** Serious and Medically Attended Adverse Events (SAEs) From Day 0 Up to Day 120 **eTable 8.** Serious and Medically Attended Adverse Events by MedDRA System Organ Class During the Study, in Randomized Participants Who Received at Least One Dose **eTable 9.** The Number Needed to Vaccinate in Cohort 1 and Cohort 2 **eTable 10.** Reason for Unmet Inclusion Criteria in Screened Participants **eFigure 4.** Percentage of Different SARS-CoV-2 Variants of Concern During the Study
eReferences

Supplement 3. **Nonauthor Collaborators.** The Soberana Study Group

Supplement 4.
Data Sharing Statement

## References

[1] Acosta-Coley I, Cervantes-Ceballos L, Tejeda-Benítez L, . Vaccines platforms and COVID-19: what you need to know. Trop Dis Travel Med Vaccines. 2022;8(1):20. doi:10.1186/s40794-022-00176-4 35965345 PMC9537331

[2] Valdes-Balbin Y, Santana-Mederos D, Quintero L, . SARS-CoV-2 RBD-tetanus toxoid conjugate vaccine induces a strong neutralizing immunity in preclinical studies. ACS Chem Biol. 2021;16(7):1223-1233. doi:10.1021/acschembio.1c00272 34219448

[3] Santana-Mederos D, Perez-Nicado R, Climent Y, . A COVID-19 vaccine candidate composed of the SARS-CoV-2 RBD dimer and *Neisseria meningitidis* outer membrane vesicles. RSC Chem Biol. 2021;3(2):242-249. doi:10.1039/D1CB00200G 35360883 PMC8826971

[4] Toledo-Romaní ME, García-Carmenate M, Valenzuela-Silva C, ; Soberana Phase 3 team. Safety and efficacy of the two doses conjugated protein-based Soberana-02 COVID-19 vaccine and of a heterologous three-dose combination with Soberana-Plus: a double-blind, randomised, placebo-controlled phase 3 clinical trial. Lancet Reg Health Am. 2023;18:100423. doi:10.1016/j.lana.2022.100423 36618081 PMC9803910

[5] Toledo-Romani ME, García-Carmenate M, Verdecia-Sánchez L, ; SOBERANA Research Group. Safety and immunogenicity of anti-SARS-CoV-2 heterologous scheme with SOBERANA 02 and SOBERANA Plus vaccines: Phase IIb clinical trial in adults. Med (N Y). 2022;3(11):760-773.e5. doi:10.1016/j.medj.2022.08.001 35998623 PMC9359498

[6] Eugenia-Toledo-Romaní M, Verdecia-Sánchez L, Rodríguez-González M, ; Soberana Research Group. Safety and immunogenicity of anti-SARS CoV-2 vaccine Soberana 02 in homologous or heterologous scheme: Open label phase I and phase IIa clinical trials. Vaccine. 2022;40(31):4220-4230. doi:10.1016/j.vaccine.2022.05.082 35691871 PMC9167831

[7] Ochoa-Azze R, Chang-Monteagudo A, Climent-Ruiz Y, . Safety and immunogenicity of the FINLAY-FR-1A vaccine in COVID-19 convalescent participants: an open-label phase 2a and double-blind, randomised, placebo-controlled, phase 2b, seamless, clinical trial. Lancet Respir Med. 2022;10(8):785-795. doi:10.1016/S2213-2600(22)00100-X 35691295 PMC9183216

[8] Chang-Monteagudo A, Ochoa-Azze R, Climent-Ruiz Y, . A single dose of SARS-CoV-2 FINLAY-FR-1A vaccine enhances neutralization response in COVID-19 convalescents, with a very good safety profile: an open-label phase 1 clinical trial. Lancet Reg Health Am. 2021;4:100079. doi:10.1016/j.lana.2021.100079 34541571 PMC8442527

[9] Sharun K, Singh R, Dhama K. Oxford-AstraZeneca COVID-19 vaccine (AZD1222) is ideal for resource-constrained low- and middle-income countries. Ann Med Surg (Lond). 2021;65:102264. doi:10.1016/j.amsu.2021.102264 33815783 PMC8010342

[10] Pollet J, Chen W-H, Strych U. Recombinant protein vaccines, a proven approach against coronavirus pandemics. Adv Drug Deliv Rev. 2021;170:71-82. doi:10.1016/j.addr.2021.01.001 33421475 PMC7788321

[11] Schulz KF, Altman DG, Moher D. CONSORT 2010 statement: updated guidelines for reporting parallel group randomised trials. J Pharmacol Pharmacother. 2010;1(2):100-107. doi:10.4103/0976-500X.72352 21350618 PMC3043330

[12] World Medical Association. World Medical Association Declaration of Helsinki. Ethical principles for medical research involving human subjects. Bull World Health Organ. 2001;79(4):373-374. 11357217 PMC2566407

[13] Bewley KR, Coombes NS, Gagnon L, . Quantification of SARS-CoV-2 neutralizing antibody by wild-type plaque reduction neutralization, microneutralization and pseudotyped virus neutralization assays. Nat Protoc. 2021;16(6):3114-3140. doi:10.1038/s41596-021-00536-y 33893470

[14] Salehi-Vaziri M, Jalali T, Farahmand B, . Clinical characteristics of SARS-CoV-2 by re-infection vs. reactivation: a case series from Iran. Eur J Clin Microbiol Infect Dis. 2021;40(8):1713-1719. doi:10.1007/s10096-021-04221-6 33738620 PMC7972329

[15] Nextclade. Clade assignment, mutation calling, and sequence quality checks. Accessed October 7, 2022. https://clades.nextstrain.org

[16] Baden LR, El Sahly HM, Essink B, ; COVE Study Group. Efficacy and safety of the mRNA-1273 SARS-CoV-2 vaccine. N Engl J Med. 2021;384(5):403-416. doi:10.1056/NEJMoa2035389 33378609 PMC7787219

[17] Ella R, Reddy S, Blackwelder W, ; COVAXIN Study Group. Efficacy, safety, and lot-to-lot immunogenicity of an inactivated SARS-CoV-2 vaccine (BBV152): interim results of a randomised, double-blind, controlled, phase 3 trial. Lancet. 2021;398(10317):2173-2184. doi:10.1016/S0140-6736(21)02000-6 34774196 PMC8584828

[18] Hotez PJ, Bottazzi ME. Whole inactivated virus and protein-based COVID-19 vaccines. Annu Rev Med. 2022;73:55-64. doi:10.1146/annurev-med-042420-113212 34637324

[19] Wouters OJ, Shadlen KC, Salcher-Konrad M, . Challenges in ensuring global access to COVID-19 vaccines: production, affordability, allocation, and deployment. Lancet. 2021;397(10278):1023-1034. doi:10.1016/S0140-6736(21)00306-8 33587887 PMC7906643

[20] Dagan N, Barda N, Kepten E, . BNT162b2 mRNA Covid-19 vaccine in a nationwide mass vaccination setting. N Engl J Med. 2021;384(15):1412-1423. doi:10.1056/NEJMoa2101765 33626250 PMC7944975

[21] Bengtsson KL, Song H, Stertman L, . Matrix-M adjuvant enhances antibody, cellular and protective immune responses of a Zaire Ebola/Makona virus glycoprotein (GP) nanoparticle vaccine in mice. Vaccine. 2016;34(16):1927-1935. doi:10.1016/j.vaccine.2016.02.033 26921779

[22] Guebre-Xabier M, Patel N, Tian J-H, . NVX-CoV2373 vaccine protects cynomolgus macaque upper and lower airways against SARS-CoV-2 challenge. Vaccine. 2020;38(50):7892-7896. doi:10.1016/j.vaccine.2020.10.064 33139139 PMC7584426

[23] Keech C, Albert G, Cho I, . Phase 1–2 trial of a SARS-CoV-2 recombinant spike protein nanoparticle vaccine. N Engl J Med. 2020;383(24):2320-2332. doi:10.1056/NEJMoa2026920 32877576 PMC7494251

[24] Shinde V, Bhikha S, Hoosain Z, ; 2019nCoV-501 Study Group. Efficacy of NVX-CoV2373 Covid-19 vaccine against the B.1.351 variant. N Engl J Med. 2021;384(20):1899-1909. doi:10.1056/NEJMoa2103055 33951374 PMC8091623

[25] Yang S, Li Y, Dai L, . Safety and immunogenicity of a recombinant tandem-repeat dimeric RBD-based protein subunit vaccine (ZF2001) against COVID-19 in adults: two randomised, double-blind, placebo-controlled, phase 1 and 2 trials. Lancet Infect Dis. 2021;21(8):1107-1119. doi:10.1016/S1473-3099(21)00127-4 33773111 PMC7990482

[26] Bian L, Gao Q, Gao F, . Impact of the Delta variant on vaccine efficacy and response strategies. Expert Rev Vaccines. 2021;20(10):1201-1209. doi:10.1080/14760584.2021.1976153 34488546 PMC8442750

[27] Tregoning JS, Flight KE, Higham SL, Wang Z, Pierce BF. Progress of the COVID-19 vaccine effort: viruses, vaccines and variants versus efficacy, effectiveness and escape. Nat Rev Immunol. 2021;21(10):626-636. doi:10.1038/s41577-021-00592-1 34373623 PMC8351583

[28] Lopez Bernal J, Andrews N, Gower C, . Effectiveness of Covid-19 vaccines against the B.1.617.2 (Delta) variant. N Engl J Med. 2021;385(7):585-594. doi:10.1056/NEJMoa2108891 34289274 PMC8314739

[29] Tang P, Hasan MR, Chemaitelly H, . BNT162b2 and mRNA-1273 COVID-19 vaccine effectiveness against the SARS-CoV-2 Delta variant in Qatar. Nat Med. 2021;27(12):2136-2143. doi:10.1038/s41591-021-01583-4 34728831

[30] Hart R. Pfizer Shot just 39% effective against Delta infection, but largely prevents severe illness, Israel study suggests. December 21, 2021. Accessed September 15, 2022. https://www.forbes.com/sites/roberthart/2021/07/23/pfizer-shot-just-39-effective-against-delta-infection-but-largely-prevents-severe-illness-israel-study-suggests/?sh=3a7b6b97584f

[31] Puranik A, Lenehan PJ, Silvert E, . Comparison of two highly-effective mRNA vaccines for COVID-19 during periods of Alpha and Delta variant prevalence. medRxiv:2021.08.06.21261707. doi:10.1101/2021.08.06.21261707

[32] Bravo L, Smolenov I, Han HH, . Efficacy of the adjuvanted subunit protein COVID-19 vaccine, SCB-2019: a phase 2 and 3 multicentre, double-blind, randomised, placebo-controlled trial. Lancet. 2022;399(10323):461-472. doi:10.1016/S0140-6736(22)00055-1 35065705 PMC8776284

