# Supplementary material for: Efficacy and Safety of a Protein-Based SARS-CoV-2 Vaccine: A Randomized Clinical Trial
Source: JAMA Netw Open. 2023 May 3;6(5):e2310302. doi: 10.1001/jamanetworkopen.2023.10302 (PMC10157429; doi:10.1001/jamanetworkopen.2023.10302)
Supplement: Supplement 1. — Trial Protocol [file jamanetwopen-e2310302-s001.pdf]

Clinical Trial Protocol

Efficacy, safety, and immunogenicity of SOBERANA recombinant vaccine (product of Finlay Institute) based on RBD protein subunit of SARS-CoV-2 in a 2-dose regimen with and without a booster dose: a double-blind, randomized, placebo-controlled phase III clinical trial in the Iranian population of 18-80 years

**Clinical Trial registration code**	IRCT20210303050558N1
**Clinical ethics code**	IR. NREC.1400.001
**Study Number**<br>(CTA number from Iran's FDA organization)	D/665/1960 Dated 24 April 2021

Edition 2.0

07 November 2021

Table of content

Contents

Funding ... 5

Sponsor ... 5

Principal investigator (PI) ... 5

University Investigators (CoPIs) ... 5

Representative of Sponsor ... 5

Background ... 7

Objectives ... 8

1. Primary Objectives ... 8

2. Secondary Objectives ... 8

Methods ... 9

1. Study Design ... 9

1.1. Overall Design ... 9

1.2 Justification for Injection of Doses on days 0, 28 and 56 ... 9

2. Study population ... 9

2.1 Number of participants ... 9

2.2 Inclusion Criteria ... 9

2.3 Exclusion Criteria ... 10

2.4 Criteria to Prohibit the Second/Third Dose During the Study (Exit criteria): ... 11

2.6 Criteria to hold the study ... 12

3. Interventions ... 12

C1: Two-dose Scheme ... 13

C2: Three-dose Scheme ... 13

3.1 Patient Recruitment ... 13

3.2 Bias Minimizing ... 14

3.3 Reasons for unmasking UUIDs 15

4. Study Procedures and Assessments 16

4.1 Efficacy Assessment 16

4.2 Immunogenicity Assessment 17

4.3 Safety Assessment..17

5. Vaccination sites 19

6. Statistical Methods 20

6.1 Sample Size 20

6.2 The Statistical Analysis Plan 21

6.3 Statistical tests..22

6.4 Working group for statistical analyses 22

6.5 Data Gathering Methods 22

6.6 Data management 23

6.7 Data monitoring plan 23

6.8 Criteria to conclude the study 24

7. Inspection and auditing..24

7.1 The Auditor's Visits..24

Outcomes...25

Primary Outcome...25

Secondary outcomes 26

The timetable of study activities 28

Table 1. Timing of activities in Cohort 1 (2-dose scheme) 28

Annexe 1. Acronyms 34

Annexe 2. Information on participating cities in each city 36

List of participating sites in the SOBERANA trial in Iran 36

Annexe 3: Description and composition of investigational products 37

Implementation of the Amendment……………………………………………………………………………………………38

presentation of the amended text……………………………………………………………………………………………38

References 41

Funding

Pasteur Institute of Iran, Tehran, Iran

Sponsor

Pasteur Institute of Iran

Principal investigator (PI)

Dr. Hamid Emadi-Koochak

Affiliation: Dept. of Infectious Diseases, Imam Khomeini Hospital, Tehran University of Medical Sciences
Address: Dept. of Infectious Diseases, Imam Khomeini Hospital Complex, East Bagher Khan St., Chamran Highway, Tehran, I.R. Iran
E-mail: emadiham@tums.ac.ir

University Investigators (CoPIs)

Isfahan University of Medical Sciences: Dr. Morteza Pourahamad
Babol University of Medical Sciences: Dr. Mostafa Javanian
Hormozgan University of Medical Sciences: Dr. Hossein Farshidi
Zanjan University of Medical Sciences: Dr. Samad Ghodrati
Mazandaran University of Medical Sciences: Dr. Roya Ghasemian
Kerman University of Medical Sciences: Dr. Iman Ghasemzadeh
Hamadan University of Medical Sciences: Dr. Fariba Keramat
Yazd Shahid Saghoughi University of Medical Sciences: Dr. Mohammadreza Mirjalili

Representative of Sponsor

Dr. Alireza Biglari

Affiliation: Pasteur Institute of Iran
Address: #69, Pasteur Avenue, Postal Code: 1316943551
E-mail: biglari63@hotmail.com

Project managers

Dr. Ehsan Mostafavi
Dr. Sana Eybpoosh
Affiliation: Pasteur Institute of Iran

DSMB Committee

1) Dr. Seyed Mahmoud Masiha Hashemi (Immunologist): Voting member
2) Dr. Seyed Saeid Hashemi Nazari (Epidemiologist): Voting member
3) Dr. Ali Dehghani Delshadi (Infectious Disease Specialist): Voting member
4) Dr. Seyed Alireza Naji (Virologist): Voting member
5) Dr. Hossein Khalili Afousi (Clinical Pharmacist): Voting member
6) Dr. Amir-Hasan Zarnani (Immunologist): Voting member
7) Dr. Majid Mokhrtari (Pulamny Dis. Specialist): Voting member
8) Dr. Ghobad Moradi (Epidemiologist): Voting member
9) Dr. Mohammad Mehdi Gouya (Representative of Iranian CDC): Non-voting member
10) Dr. Reza Mosaed (Representative of FDA): Non-voting member
11) Dr. Fariba Asghari (Representative of the National Ethics Committee): Non-Voting member

Study monitoring and audit team

1) Dr. Ali Khamesipour: Tehran University of Medical Sciences
2) Dr. Amir Javadi: Qazvin University of Medical Sciences
3) Dr. Seyed Ebrahim Eskandari: Tehran University of Medical Sciences
4) Dr. Farhad Handjani: Shiraz University of Medical Sciences

Adverse Event Evaluation Committee

1) Dr. Seyed Mohsen Zahraei: Center for Communicable Disease Control, Ministry of Health
2) Dr. Malahat Khalili: Kerman University of Medical Sciences
3) Dr. Katayoun Tayeri: CDC, Ministry of Health
4) Dr. Ali Asadollahi-Amin: Tehran University of Medical Science
5) Dr. Mostafa Javanian: Babol University of Medical Sciences
6) Dr. Fariba Keramat: Hamadan University of Medical Sciences
7) Dr. Samad Ghodrati: Zanjan University of Medical Sciences
8) Dr. Ali Khamesipour: Tehran University of Medical Sciences
9) Dr. Roya Ghasemian: Mazandaran University of Medical Sciences

Background

At the end of December 2019, China reported several pneumonia cases of unknown aetiology in Wuhan. The disease caused by Severe Acute Respiratory Syndrome Coronavirus 2 (SARS-CoV-2), a member of the coronaviridae family, was named COVID-19 and made a rapidly growing epidemic throughout the world, reaching 186 countries to date.

SARS-CoV-2 has four structural proteins: spike (S), envelope (E), membrane (M), and nucleocapsid (N). Protein S mediates the virus entry to the host cell through its Receptor-Binding Domain (RBD) attachment to the host cell receptor (ACE2) and therefore is the target of neutralizing antibodies. These features make the RBD a good candidate for S-protein-based COVID-19 vaccines.

The Finlay Vaccine Institute, a leader in Cuban health technology, designed the FINLAY-FR-2 (SOBERANA 02) vaccine for emergency use against SARS-CoV-2. This vaccine contains an injectable suspension containing the S protein's receptor-binding domain (RBD), conjugated to tetanus toxoid and adsorbed on aluminum hydroxide gel.

Finlay Institute has recruited this conjugation technology to produce various vaccines like the *Haemophilus influenzae* type B vaccine (Quimi-Hib) within the past 15 years.

Conjugate vaccines can induce a robust immune response against bacterial infections like *Haemophilus influenzae, Neisseria meningitidis*, and *Streptococcus pneumonia* by linking bacterial polysaccharides to a carrier protein. The immune system normally recognizes polysaccharide chains as T-independent (TI) antigens, which means B-cell proliferation and antibody secretion happen in the absence of T cells. The conjugated bacterial polysaccharides - carrier proteins, d*eceive the immune system into identifying the complex as a T-dependent antigen which means the induction of B-cell proliferation* requires T-cell help. As a result, a long-lasting immune response will be obtained by forming memory B and T cells. Compared with unconjugated vaccines, IgG antibodies are more produced and have higher avidity to the conjugate antigen, representing a longer immunity against bacterial infection. In addition,

children<2 years are non-responsive to most unconjugated vaccines due to their immature immune systems; however, conjugate vaccines are highly effective in this group of age.

Additionally, unconjugated polysaccharide-based vaccines require approximately 5-10 times more antigens than their conjugated counterparts to reach the determined immunity, so conjugate vaccines are probably less reactogenic due to their lower antigen levels.

Preclinical evaluations of the SOBERANA-02 vaccine in animals showed immunogenicity, high anti-RBD IgG antibody titers, and blocked RBD/ACE2 interaction. Furthermore, Repeat-dose toxicity studies showed safety, tolerability and no toxicity.

Phases 1 and 2 of the study in Cuba also showed these vaccine candidates' safety, reactogenicity, and immunogenicity. We will conduct a randomized, double-blind, placebo-controlled phase 3 trial of Soberna-02 and SOBERANA Plus (products manufactured by Finlay Institute of Cuba) to evaluate the efficacy and safety of these candidates in Iranian adults.

Objectives

1. Primary Objectives

- To determine the efficacy of protein conjugated vaccine/ protein conjugated vaccine + third dose to prevent symptomatic COVID-19 in 18–80-year-old Iranians.

2. Secondary Objectives

- To determine the efficacy of protein conjugated vaccine/ protein conjugated vaccine + third dose to prevent severe forms of COVID-19 in 18–80-year-old Iranians.
- To determine the efficacy of protein conjugated vaccine/ protein conjugated vaccine + third dose to prevent COVID-19-related death in 18–80-year-old Iranians.
- To determine the immunogenicity of protein conjugated vaccine/ protein conjugated vaccine + third dose in the 18–80-year-old Iranians.
- To determine the safety of protein conjugated vaccine/ protein conjugated vaccine + third dose in 18–80-year-old Iranians.

Methods

1. Study Design

1.1. Overall Design

This study is a multicentric, randomized, double-blind, placebo-controlled phase-3 trial to evaluate the efficacy, safety, and immunogenicity of the SOBERANA-02 (25µg) and SOBERANA Plus (50µg) in healthy adults or adults with stable underlying diseases.

The study consists of two cohorts to evaluate the efficacy, immunogenicity and safety of two vaccination regimens:

- Cohort 1: covers a 2-dose schedule of SOBERANA-02 (25µg), 28 days apart
- Cohort 2: as a 3dose schedule (2 doses of SOBERANA-02 [25µg] plus the third dose of SOBERANA-plus) 28 days apart

1.2 Justification for Injection of Doses on days 0, 28 and 56

The injection scheme is determined based on preclinical studies on animals. Knowing the immune system performance usually for vaccines based on prime-boost strategy, the interval between the two doses is at least three weeks. For example, the interval between two doses of Pfizer, Moderna, AstraZeneca, and Sputnik V is 21, 28, 28, and 21 days, respectively. For the SOBERANA candidate vaccine, the interval between doses is also set at 28 days.

2. Study population

2.1 Number of participants

The estimated sample size is 24,000 (allocation ratio of 4:1 for vaccine vs. placebo groups).

- The C1 cohort includes 18,000 volunteers who will receive two doses of SOBERANA-02 on days 0 and 28 in 6 cities (Bandar Abbas, Kerman, Isfahan, Hamadan, Babol, and Sari)
- The C2 cohort includes 6,000 volunteers who will receive two doses of SOBERANA-02 (days 0 & 28) plus a third dose of SOBERANA-plus on day 56 in two cities (Zanjan and Yazd)

The selected areas based on the number of doses and laboratory assays are detailed in Box 1.

2.2 Inclusion Criteria

- Male or female aged 18 -80 years old

o Participants who can follow vaccination schedules, visits, laboratory tests and other study procedures.

o Participants with healthy conditions or/with a controlled medical condition(s) that is under control.

o Having Iranian nationality
o Residents of the cities of the study

2.3 Exclusion Criteria

- Previous receipt of any COVID-19 vaccines
- Current clinically or lab-confirmed COVID-19 at the time of enrolment
- History of tetanus toxoid vaccination within three months before the current study
- Pregnant or breast-feeding women or those who intend to get pregnant within three months after the last vaccine shot
- History of receiving blood or its products such as immunoglobulin within the past three months
- Uncontrolled hypertension (systolic blood pressure>140 mmHg, or diastolic blood pressure > 90 mmHg)
- Type 2 diabetes mellitus (HbA1C > 7.5%)
- Chronic renal disease (GFR < 30 CC/min)
- Chronic liver disease (AST≥ 100 U/L, ALT≥ 150 U/L)
- Uncontrolled asthma (asthmatic attack within the past three months)
- Severe allergic reaction to any vaccines (anaphylaxis)
- Cigarette smoking (more than 20 per day for more than 20 years)
- Contraindication for muscular injections due to coagulation disorders
- Mental diseases
- Any coagulation disorders that contraindicate intramuscular injections
- Using immunosuppressive medicines like nasal and oral steroids (topical steroids are not exclusion criteria), cytostatic agents, interferon, inmunoferon, transfer factor, Biomodulin T, Gamma globulin, Levamisole, Heberferon, Thymosin, and other immunomodulatory drugs since one month before vaccination
- Fever or any acute infectious disease within seven days before vaccination
- Tattoos on the deltoid muscle of both arms.

- Those in phase 1 of the national plan for establishing and expanding COVID-19 vaccination.[1]

Note

Up to 30% of volunteers with the following conditions can enter the study:

- Obesity (BMI >30 kg/m2)
- Controlled hypertension (both self-declared and on-site systolic blood pressure <140 mmHg and diastolic pressure< 90 mmHg, the volunteer will be included in the study despite using antihypertensive drugs)
- Chronic renal disease (based on the lab test result in the past six months): GFR: 30-60 CC/min; GFR < 60 will not be included in the study.
- Chronic liver disease that is < 5 folds to the normal range (based on the lab test results in the past six months): AST< 100 U/L, ALT< 150 U/L
- Type 2 diabetes mellitus (based on the lab test result in the past six months): HbA1C < 7.5
- Chronic obstructive pulmonary disease (COPD)
- Controlled asthma (no asthmatic attack in the past three months)
- A history of any malignancy or cancer
- Ischemic heart disease

2.4 Criteria to Prohibit the Second/Third Dose During the Study (Exit criteria):

- A diagnosis of COVID-19 with PCR test after the last dose of injection
- Severe fever (axillary temperature ≥ 38°C) for three days or allergic reaction to the previous dose of the vaccine
- SAEs following the previous shot
- Vaccination with diphtheria/tetanus vaccine between the two doses of the candidate vaccine
- Volunteer being pregnant

[1] Based on the national plan to establish and expand COVID-19 vaccination, everyone in phase 1 of vaccination priority will receive the vaccine in the next few weeks. These individuals are ineligible to participate in this study if they are in the following groups: All employees who provide first-line care to COVID-19 patients at high risk of contracting and transmitting the infection, including healthcare personnel in both the public and private sectors, the country's laboratory, faculty and staff of universities as well as the Ministry of Health, Treatment and Medical Education; People at risk of hospitalization and death due to COVID-19, including nursing home residents and nursing home staff, Veterans esp. veterans over 50% and chemical respiratory veterans living in veterans' care centers, and mentally and physically disabled people living in these centers.
A restriction applies to health workers currently in the first phase of national vaccination priorities, despite receiving general vaccinations with approved vaccines. If they wish to enroll in this trial and enter the study, there is a prohibition against it.

- A diagnosis of COVID-19 with PCR test after the last dose of injection Severe fever (axillary temperature ≥ 38°C) for three days or severe allergic reaction (anaphylaxis) to the previous dose of the vaccine

- Receiving drugs affecting the immune system between two doses of the vaccine

- Any disease that affects a candidate's immunity level between two injections, based on the diagnosis of a medical professional

2.5 Criteria for rescheduling the vaccine (after solving the problem)

- having fever or acute illness within 7 days before or on the day of injection
- having high blood pressure (systolic pressure more than 140 or diastolic pressure more than 90 mm Hg)

Note: The reason/s for every volunteer's exit from the study will be recorded clearly in the eCRF while his/her safety information will be gathered till the end of the study and reflected in the final and follow-up report

2.6 Criteria to hold the study

· Recognition of executive or planning issues in the study, which need investigation

· Detection of potential safety issues for participants by DSMB

· Safety issues diagnosed by the sponsor based on the DSMB report or independently

· Suspected Unexpected Serious Adverse Reactions (SUSAR): If a SUSAR occurs, the study stops temporarily as of investigation team concludes the causality of SAR and decides to stop or continue the study

· A request be sent by the National Committee for Ethics in Biomedical Research to hold the study

· A request be sent by the NRA (national regulatory authority, FDA) to hold the study

3. Interventions

The participants will receive the vaccine candidates according to two schemes (cohorts).

C1: Two-dose Scheme

Intervention group: Intramuscular injection (Deltoid muscle) of 25 µg SOBERANA-02 vaccine on days 0 and 28

Placebo group: Intramuscular injection of placebo on days 0 and 28

C2: Three-dose Scheme

Intervention group: Intramuscular injection (Deltoid muscle) of 25 µg SOBERANA-02 vaccine on days 0 and 28 as well as a third dose with SOBERANA-plus on day 56

Placebo group: Intramuscular injection of placebo on days 0, 28 and 56

3.1 Patient Recruitment

3.1.1 First stage*:* We will invite the public via social networks, text messages, and media. Our trained staff will define the objectives of the study and inclusion criteria for participants and make sure that everyone understands them. Further information will be available on the recruiting website. Participants can register on the website after assessing the eligibility criteria. The required registration items will be the city name, national ID number, phone number, and history of underlying disease(s). Healthy people with chronic conditions that are under control (diabetes, liver or renal disease, and hypertension) can participate in the study after a complete examination by the study physician and laboratory results in the following range:

- Chronic renal disease (GFR > 30 CC/min calculated based on the blood creatinine level)
- Chronic liver disease (AST< 100 U/L, ALT< 150 U/L)
- Type 2 diabetes mellitus (HbA1C < 7.5%)
- Hypertension (Systolic blood pressure< 140 mmHg and diastolic blood pressure <90 mmHg on medication based on self-declaration and on-site vital sign assessment)

3.1.2 Second stage*:* The eligible volunteers will receive preliminary approval by a text message containing the appointment date, time, and address of the vaccination

3.1.3 Third stage (visit 1): In case of any ambiguity about the situation, volunteers can ask further questions from research physicians.

After taking the informed consent in two copies, the physician will examine the volunteers and order further screening or laboratory analyses. All consenting participants will be screened for underlying chronic conditions and COVID-19-related clinical symptoms. Then volunteers will receive a unique randomization ID that helps them to check their vaccination status until the end of the study.

3.2 Bias Minimizing

3.2.1 Randomization (allocation)

To allocate the participants, we designed a specific software to calculate the sample and each block size, the number of recruiting centers, and the intervention arms ratio. We will use the stratified block randomization method to equally allocate subjects from the same strata to each group.

The total sample size in two cohorts is considered 24000 divided into eight cities (i.e., 3000 volunteers in each vaccination site). Each block includes 20 vaccine vials and five placebo vials randomly distributed within the block. Each vial has a label with a universally unique ID (UUID) and a one-digit code for the designated city, while the allocated blocks to each city are labelled with numbers from 1 through 140. Therefore, the volunteer UUID includes five digits, the first one is set for the city (vaccination center), and the next four digits depict a number from 1 through 3000, so all those UUIDs are unique in this study.

Knowing the sample size in each center and the block size, the software computes the necessary number of blocks and constructs a random chain for each block based on the algorithm suggested by Magalhães et al. (9). Briefly, in this method, at first, a list of intervention and placebo UUIDs is constructed. For example, in a 6-member block and 1:1 ratio of groups, the intervention list is organized as [0,0,0,1,1,1], then the list indices are randomly incorporated, and the list is organized based on the new order of indices. The indices are sorted based on a randomized process, in which in each step, one of the indices is selected via homogeneous distribution and added to the final sort-out and omitted from the non-selected indices. This process is repeated for each block. The randomization program is written in Python 3.8.2. For the construction of random numbers, we applied the Mersenne Twister algorithm, and the seed of randomization in that program is determined with a computer clock when the program runs. Every report file is named using date, and for validation purposes, the checksum of its content is calculated with the MD5 method and is kept in a separate file.

Allocations and trial management were performed using an online platform. The vaccine and placebo vials were identical and indistinguishable in appearance.

3.2.2 The process of assigning UUID to the subjects

The orders of universally unique identifiers (UUID) are in the software and are inaccessible to the researcher. Study information is collected and managed through a web system developed by the knowledge-based company "Vista Technology Ideas Group." One copy of the randomly allocated list is given to Iran’s FDA. The UUIDs are printed on adhesive labels. Those labels are attached to each volunteer’s vaccine vials. After receiving informed consent and preliminary examination, eCRF will be completed for the volunteer. After completing eCRF and if the volunteer is considered “eligible for the study,” a random identifier will be allocated to him/her and recorded. For the whole duration of the study, the UUID will be applied to all planned processes. As mentioned before, the assigned UUID is a 5-digit number the first digit is the city code, and the remaining four digits could be a number from 0001 to 3000.

3.2.3 Allocation concealment

Allocation concealment will be using central allocation. A central team will perform subject assignments. After randomization, each participant will receive one out of 25 codes in the assigned block. The codes will not be clear for the participants and study teams at each site.

3.2.4 Blinding

The UUIDs will be delivered to a group in charge of labelling the vials. None of the people in the vial-labelling group would have a role in recruiting study subjects or vaccine injection. They do not have any contact with the recruitment and vaccination site staff either. Thus, when sending the vial blocks to vaccination sites, no investigator can differentiate the vaccines from placebo vials. The suspensions in the vaccine and placebo vials are milky white and similar in colour and clarity. Moreover, vaccine and placebo vials are offered in similar appearance, they are inseparable, and packaging and will be placed in boxes of 25 vials on each box, the block number and serial numbers of vaccines/placebos are written. This process yields in the blindness of the study participants, vaccinators, investigators, and monitors.

Vaccinators will check the assigned UUID to each volunteer with the UUID labelled on the vial. All used vials in the study will be archived and kept secure.

3.3 Reasons for unmasking UUIDs

In case of any emergency (such as the occurrence of SAEs), unmasking will be undertaken urgently. Just one investigator is authorized for urgent unmasking with the aid of the developed

online platform. The Co-PI is fully responsible for unmasking the volunteer's treatment group. The health and safety of volunteers is the most important consideration in Co-PI's decision-making about this issue. For this purpose, Co-PI will enter the electronic platform under emergencies, log into the electronic platform, open his page and send his request for opening the subject's UUID with a justification of the reasons to do that. This request will be submitted to the PI and the investigator authorized to unmask under emergency conditions. The last one will log into his page on the electronic platform and concur with the unmasking, then let the CoPI know the intervention group of the volunteer. All information on the unmasking will be recorded within the electronic platform. Afterwards, related sheets to inform the sponsor, national ethics committee and the national regulatory authority (FDA) will be filled out and submitted. The investigator in charge of unmasking will inform the sponsor for permission before opening the UUID unless delay threatens the affected volunteer. The date and reason for decoding UUID will be recorded in eCRF. Elderly people over 65 years of age, who are at high risk of contracting severe forms of the disease, will be proactively contacted if they are prioritized to receive the vaccine according to the national vaccination document and the announced policies of the National Corona Headquarters, and the intervention group they will be informed and in the case of the control group, they will be informed about providing them access to the vaccine of the national vaccination program.

In the case of other groups that are prioritized to receive vaccines according to the national vaccination document, the policies announced by the National Corona Headquarters and the current programs of the country, upon their request, the code will be decoded and their treatment group will be informed.

If the candidate has received one or two doses of the current vaccine and is prioritized for general vaccination with other vaccines, it is recommended that he remain in the current study and perform subsequent injections with the vaccine. However, if they want to withdraw from the study with their consent and inject another vaccine, the program administrator is only responsible for the vaccine received in this program and the consequences of the change. The vaccine will be their responsibility and the financial sponsor of the program (sponsor) will not be responsible for it.

4. Study Procedures and Assessments

4.1 Efficacy Assessment

The vaccine efficacy will be considered in terms of the prevention of symptomatic COVID-19 infection (as the primary outcome), as well as severe COVID-19 infection and COVID-19-related

death (as secondary outcomes), all of which will be considered in efficacy analysis if they occurred from 14 days after the second/third dose onward.

4.1.1 Case Definition

o **Symptomatic COVID-19 infection** is defined as those presenting with two or more of the following symptoms lasting for over 24 hours confirmed with a positive RT-PCR testing: Fever (temperature ≥38°C), chills, new cough, sore throat, nasal congestion, fatigue, muscle or body pain, headache, nausea or vomiting, diarrhea, loss of smell or taste, or at least one respiratory sign or symptom (i.e., cough, shortness of breath, or clinical or radiographic evidence of pneumonia).

o **Severe COVID-19 infection** will be considered as O2 saturation level ≤90%, evidence of lower respiratory disease (e.g., shortness of breath, chest pain or chest tightness) with or without fever ≥38°C during clinical assessment or imaging, tachypnea (i.e., respiration rate >30 breaths/min), increased P(A-a) O2 gradient, lung infiltration >50% in CT-scans, PaO2/FiO2 <300 mmHg, or acute worsening of respiratory symptoms especially dyspnea, respiratory failure, septic shock, and/or multiple organ dysfunction, hospitalization or death due to COVID-19.

o **COVID-19-related Death** will be considered based on the WHO definition: deaths not attributable to another cause (e.g., trauma). There should not be a recovery interval between COVID-19 infection and death.

4.2 Immunogenicity Assessment

To assess humoral immunity, Anti-S1(including RBD) IgG antibodies will be measured by ELISA in a subgroup of volunteers in Babol and Sari (C1) and Zanjan (C2) before the first dose and 28 days after the last dose. In addition, on Days 5 and 28, an extra antibody measurement will be done on a random subset of 900 volunteers in Babol and Sari (30% of volunteers in these cities). Limiting these extra measurements in two cities is to minimize the blood sampling and ease the logistic issues. Neutralizing antibodies will be measured by the Virus Neutralization test (VNT) in a proposition of participants (100 individuals).

To assess Cell-Mediated Immunity (CMI), Interferon Gamma Release Assay (IGRA) will be performed on at least 130 participants from Babol or Sari.

4.3 Safety Assessment

Whether solicited or unsolicited, the local and systemic adverse events (AEs) will be recorded in diary forms by the volunteers. The AEs will be recorded from the first dose (Day 0) until five months after the last dose in both cohorts.

4.3.1 Adverse Events (AEs)

The AE is defined as any untoward medical events in participants that are temporally associated with the use of study interventions, whether or not considered related to vaccine/placebo. An AE can vary from clinical manifestations to abnormal test results.

After each injection, systemic and local pre-defined AEs will be registered using an online platform within three days. Solicited local AEs occur in the injection site and include pain, redness, swelling, induration, and warmth. Solicited systemic AEs are fever, chills, nausea, vomiting, malaise, fatigue, headache, diarrhea, pain in extremities, and rashes. Participants will record all these events using an online platform within three days after receiving each dose of vaccine or placebo. If any volunteer will not complete the diary, an active follow-up will be done on day four. Moreover, other unspecified symptoms (unsolicited AEs) will be followed up 28 days after each injection.

Serious Adverse Events (SAEs) are defined as any event which leads to a dangerous situation, hospitalization, longer duration of hospital stays, serious/permanent disability, congenital anomalies, requires a medical or surgical intervention to prevent permanent injury, or culminates in death. These events will be monitored up to five months after the last dosage of vaccine/placebo.

Medically Attended AEs (MAAEs) are adverse events after taking intervention that requires presentation to a health care provider, such as a general practitioner or emergency room visit for physical examinations. These events will be monitored up to five months after the last dosage of vaccine/placebo.

For monitoring and managing of AEs, the following measures will be performed:

- Active monitoring of participants for adverse events for half an hour after receiving each dose at vaccination sites.

- An Emergency medical services (EMS) ambulance will stand with three paramedics. There is an attending emergency medicine specialist with all first-aid and resuscitation equipment in place

All arrangements and coordination have been made with a supporting hospital in each city to transfer and visit participants who might suffer SAEs. In case of any SAE, the emergency specialists of the study will coordinate with the hospital an appointment for participants. We will provide 24-hour free-of-charge medical services in these hospitals for participants during the study.

- The solicited AEs (local and systemic) are recorded in online diary forms up to 72 hours after receiving each dose by volunteers. If any volunteer does not complete the diary, an active follow-up will be done on day fourth. During this period, medical services and consultations, and hospitalization are provided to volunteers who are affected by AEs.

- Passive monitoring of unsolicited AEs from the first day until day 28th is done through spontaneous reporting by participants to call centers or go to specified hospitals.

- SAEs and MAAEs are detected passively whenever a volunteer report to the call center or consults with the hospital. These participants will be followed actively until fully recovered.

- All SAEs and MAAEs should be reported at the earliest time possible (a few days) to the sponsor and PI, who will inform DSMB to decide about unmasking and withdrawing the participants from the study.

- In case of any SAE or fatal event that needs hospitalization, the CoPIs will report the case to the Pasteur Institute of Iran via an urgent phone call, which in turn reports to FDA, and the National Ethics Committee (NEC).

4.3.2 Causality Assessment of AEs

All SAEs and MAAEs will be assessed and classified by the Adverse Events Classification Committee. We will use WHO's adverse event following immunization (AEFI) causality assessment methodology for a causality assessment of AE after injection. In this method, the cause-specific definitions provide clarity on "A. Consistent causal association to immunization" and "C. Inconsistent causal association to immunization (coincidental)." The association is considered "B. indeterminate" when adequate information on the AEFI is available, but it is not possible to assign it to either of the above categories. Moreover, cases without adequate information for causality conclusions are categorized as "D. unclassifiable."

5. Vaccination sites

We will perform the trial in vaccination sites designed and equipped by local medical Universities in 8 recruited cities (Bandar Abbas, Kerman, Yazd, Isfahan, Babol, Sari, Zanjan, and Hamadan). All vaccination sites must include the following spaces:

- A hall for instruction and signing informed consent by volunteers
- A waiting hall for volunteers

· Medical examination and screening rooms

· Blood sampling units

· Vaccinations rooms

· Post-vaccination active monitoring hall

· Emergency and Cardiopulmonary Resuscitation (CPR) unit

· An ambulance (EMS), three paramedics, and at least an emergency medicine specialist will be ready at vaccination sites to provide primary care in case of serious adverse events.

· In each city, a supporting hospital will service volunteers for probable serious adverse events (SAEs).

Recruited laboratories will test the participants for PCR, screening, and serology. The principal investigator will follow the standard operating procedure (SOP) to perform the laboratory process. Pasteur Institute of Iran will be responsible for quality control (QC) and quality assurance (QA) based on pre-defined protocols. We mentioned the address of the vaccination sites, contracting labs, and investigators of recruiting cities in Annex 2.

6. Statistical Methods

6.1 Sample Size

Separate power calculations were performed for the two cohorts. To demonstrate a clinically meaningful difference, we assumed 50% efficacy for two-dose regimens (C1) based on WHO criteria and hypothesized 70% efficacy for the three-dose regimen (C2) based on an independent Technical Support Committee (TSC) opinion that considered the immune response results observed in phases I and II trials of the 2 and 3 dose regimens. Other assumptions included an exponential distribution, minimum baseline incidence of disease in two (0.012) and three-month (0.018) follow-ups (incidence rate: 0.0055 per month), maximum dropout proportion of 10% and 20%, and a vaccine-to-placebo ratio of 4:1. Type I (a two-sided hypothesis) and type II errors of 0.05 and 0.1 were considered for sample size calculation in each cohort. The total sample size was calculated to be about 18,000 participants in C1 (14,400 in the vaccine and 3,600 in the placebo group) and 6,000 participants in C2 (4,800 in the vaccine and 1,200 in the placebo group) using Stata software.

Software command for sample size calculation

Software: Stata (v.14.2)

Cohort 1
power exponential 0.0055, hratio(0.5) power(0.9) nratio(4) fperiod(2) lossprob(0.1) losstime(2)

Cohort 2
power exponential 0.0055, hratio(0.3) power(0.9) nratio(4) fperiod(3) lossprob(0.2) losstime(3)

6.2 The Statistical Analysis Plan

6.2.1 Efficacy population

To analyze the efficacy, we will apply by "Intention to treat (ITT)" analysis.

6.2.2 Immunogenicity population

To evaluate the vaccine immunogenicity, we will use a PP analysis, through which the immunogenicity indicators, i.e., seroconversion and neutralization, will be measured in those subjects receiving all vaccine doses and compared with the placebo group.

6.2.3 Safety population

We will benefit from a "per-protocol (PP)" analysis to evaluate vaccine safety. In this approach, the solicited and unsolicited adverse events will be determined and compared between those subjects receiving at least one dose of vaccine and those receiving at least one dose of placebo.

6.2.4 Subgroups analyses

Vaccine Efficacy will be calculated among different subgroups of gender (Male and Female), ages (under and above 65), and underlying diseases (yes and no) using stratified Cox regression and logistic model for long format dataset using a working correlation matrix.

6.2.5 Analysis of efficacy

Considering the high incidence of COVID-19 in Iran and the high sample size of the study, the duration of follow-up for the efficacy analysis in C1 is estimated to be four months (3 months after the second dose) making it possible to estimate the efficacy with a significance level of α

<0.0001. The study duration for safety analysis in C1 is six months. Also, the duration of follow-up for the efficacy analysis in C2 is estimated to be six months (4 months after the third dose). Such a duration brings a possibility to estimate the efficacy with a significance level of $\alpha<0.0001$. The study duration for side effect analysis in C2 will be seven months (or five months after the third dose).

6.3 Statistical tests

We will apply the stratified Cox regression model to estimate vaccine efficacy. The efficacy will be reported using the "1-Hazard Ratio". Also, vaccine efficacy will be estimated through a logistic regression on the long person-time format dataset using a working correlation matrix. In this model, the efficacy will be reported using the "1-OR".

In each study arm (placebo vs. treatment), the frequency of adverse reactions or events will be described and estimated with 95% CI. The seroconversion frequency (cases with a four-fold rise in S antibody titer compared to the base) will also be described with 95% CI in each study arm. Furthermore, the geometric mean of the antibody titer will be calculated and reported with a 95% CI. Antibody titers between the vaccine and placebo groups will be compared with Mann-Whitney U. Also mean difference and the mean ratio will be calculated. The frequency of serious and unsolicited adverse events will be separately described and categorized system-by-system. The affected body systems are based on the classification in the "Medical Dictionary for Regulatory Activities" (MedDRA). All statistical analyses will be performed with R and Stata software (v. 16).

6.4 Working group for statistical analyses

In this RCT, an independent team will do data gathering, data cleaning, statistical analyses and interpretation of results. The members of that working group are affiliated with the Kerman University of Medical Sciences. The team consists of Bio-statisticians and Epidemiologists.

6.5 Data Gathering Methods

Data collection will be through electronic forms. Co-PIs in 8 cities are in charge of supervision of data collection. Research data will be collected and managed via a web-based platform developed by an Iranian knowledge-based company titled "Vista idea builders in Technology." This company supports the platform as well.

Each recruitment and vaccination site has its access level, which is separated from the other cities and enables operators to enter data, CoPIs to ask for data editions, and the PI to approve editions. The platform helps PI and sponsors monitor the RCT progress and make reports for DSMB and steering committees. The volunteer records its adverse events in the AEs report form till three days after each dose and hands it over to one of the designated physicians at the vaccination site. Also, it is possible to report AEs telephonically or to a designated hospital in person, which will be recorded with the volunteer's UUID in eCRF.

6.6 Data management

The PI is accountable for holding all forms and documents related to study participants during the study. After the study, all forms and documents will be submitted to the data management center at the PI's request. One copy of documents can be maintained in each study center at CoPI demand. All confidentiality principles will be followed in submitting and receiving all documents.

6.7 Data monitoring plan

As a part of the GCP of this RCT, the monitoring team will monitor the implementation, recording and reporting of the study periodically.

6.7.1 Monitoring Team Visits

6.7.1.1 Pre-trial Monitoring: The monitoring team visits each city 2-7 days before the initiation of its vaccination site activity. The visit aims to assess the capabilities of the visited city to start the study, e.g., sufficient vaccine supply, necessary documents and equipment, investigators and staff and their knowledge.

6.7.1.2 Trial Initiation Visits: Will be held one day before the initiation, and processes fluency will be checked, and necessary feedback for improvements will be provided. On the following day, the vaccination site will be officially inaugurated during a ceremony with influential figures in the city. Such a design guarantees community engagement. On the first day, the monitoring team continues to provide feedback to the site staff and address questions or any remaining gaps.

6.7.1.3 Routine Monitoring Visits: The monitoring team will visit each city at least twice during the study period in a one-month interval. The objective is to make sure the accuracy of the implementation phase is based on GCP standards.

6.7.1.4 Close-out Visit: At the end of the study in each city (3 months after the last vaccine dose), a visit will be considered to ensure an accurate archive of documents, return of study products, equipment and others.

6.8 Criteria to conclude the study

In case the following criteria are met, the study can be concluded:

- All serologic studies are performed according to timetable and test results are reported
- All volunteers' visits are done and related documents are delivered to the PI and archived
- All remaining vials are counted and given back to the sponsor
- The final study report is written

7. Inspection and auditing

As a component to establish a quality assurance process, Pasteur Institute of Iran has considered the auditing process. Auditing the study will be done by an independent person selected by the sponsor and will visit the designated cities and vaccination sites monthly without any advance notice. The purpose of the sponsor's audit is independent assessments of all activities, logistics and documents of the study to ensure that those are well corresponding with the study protocol. The audit reports are submitted to the primary research team and the sponsor.

7.1 The Auditor's Visits

1- **The scheduled visits:** The auditing team will visit study sites before the study and at monthly intervals during the study, so they guide the implementers and provide feedback to diminish possible errors and any diversion from the protocol.

2- **Ad hoc visits for specific reasons:** In case of any SAE or any report of serious issues in quality implementation in one or several vaccination sites, the auditor will visit those sites at the sponsor's request.

Box 1. Classification of Recruiting Cities Based on the Number of Doses and Laboratory Assays

C1: Two-dose of SOBERANA-02 (days 0 & 28): We will recruit a total sample size of 18,000 volunteers in Six cities of **Babol, Sari, Isfahan, Kerman, Bandar Abbas, and Hamadan** (3000 volunteers each).

1. In **Babol and Sari,** all the volunteers will get routine screening and blood draws on Days 0 and 56. A random subset of 900 volunteers (30%) will get an additional serology test on Days 5 and 28.
2. In **Isfahan, Kerman, Bandar Abbas, and Hamadan,** all the volunteers will get routine screening and blood draws without serology tests.

C2: Two-dose of SOBERANA-02 + third dose of SOBERANA-plus (Days 0, 28, & 56): We will recruit a total sample size of 6,000 volunteers in two cities of **Zanjan and Yazd** (3,000 volunteers each).

1. **Zanjan:** All the volunteers will get routine screening and blood draws during the study. A random subset of 2,000 volunteers will get additional serologic tests on days 0 and 84 (1 month after the third dose)
2. **Yazd:** All the volunteers will get routine screening and blood draws without serology tests.

Outcomes

Primary Outcome

Outcome	Definition	Measurement method	Timing of measurement
Symptomatic COVID-19-19 infection	Identification of SARS-CoV-2 RNA (two viral gene targets) in the respiratory samples of suspected cases	Real-time PCR	**C1:** From day 14th after the 2nd dose until the end of efficacy study time<br><br>**C2:** From day 14th after the 3rd dose until the end of efficacy study time

Secondary outcomes

Outcome	Definition	Measurement method	Timing of measurement
Severe COVID-19 infection	Includes severe, critical or hospitalized COVID-19 cases	Included those COVID-19 patients with O2 saturation level ≤90%, evidence of lower respiratory disease (e.g., shortness of breath, chest pain or chest tightness) with or without fever ≥38°C during clinical assessment or imaging, tachypnea (i.e., respiration rate >30 breaths/min), increased P(A-a) O2 gradient, lung infiltration >50% in CT-scans, PaO2/FiO2 <300 mmHg, or acute worsening of respiratory symptoms especially dyspnea, respiratory failure, septic shock, and/or multiple organ dysfunction, hospitalization or death due to COVID-19.	**C1:** From day 14th after the 2nd dose until the end of efficacy study time<br>**C2:** From day 14th after the 3rd dose until the end of efficacy study time
COVID-19-related death	Based on the WHO definition: The death of a COVID-19 case is not attributable to another cause (e.g., trauma). There should not be a recovery interval between COVID-19 infection and death	Based on the diagnosis of PI and Co-PIs in the cities and approval of DSMB	
Humoral immunity	At least a 4-fold rise in Anti-S1 IgG titer in serum sample compared to the baseline	ELISA	28 days month after the last vaccine shot compared to the serum titer of Day 0
Virus neutralization	Detection of antibodies which can inhibit virus replication (an antibody, which can neutralize virus infectivity)	VNT (Virus Neutralization Test)	On days 0 and 28 days after the second dose, serum samples will be taken from a subset of antibody-positive volunteers and the neutralizing antibody titer will be determined

Cell-mediated immunity (CMI)	Measuring Interferon Gamma secreted from PBMC (peripheral blood cells mononuclear cell)	Interferon Gamma Release Assay (IGRA)	In the cities of Babol and Sari, CMI will be performed
Safety	Frequency of local and systemic adverse events disaggregated by intensity (degree) and seriousness (type)	Case investigation form, Diary form, telephonic follow-up	From day 0 until 5 months after the last dose

The timetable of study activities

Table 1. Timing of activities in Cohort 1 (2-dose scheme)

**No. of visit**	**1**	**2**	**3**	**4**		
**Objective of visit**	**Screening Dose 1, Blood sampling §**	**Blood sampling from a subset of subjects§**	**Blood sampling from a subset of subjects §**	**Blood sampling §**	**End of monitoring efficacy**	**End of monitoring adverse events**
**Month**	**Month 1**			**Month 2**	**Month 4**	**Month 6**
**Day**	**0**	**5**	**28**	**56**		
**Acceptable interval (open window period)** [1]		**+ 2**	**+ 7**	**+ 7**		
Informed consent<br>Complete eCRF (demographic info, concurrent treatments, history of underlying conditions, etc.)	●					
Blood sampling to assess the previous infection of COVID-19 (antibody assay)	●					
Physical examination [2]	●		●			
Randomization	●					
**Intervention**						
Vaccine injection and active monitoring for 0.5 hours after the shot	●		●			
**Evaluation of vaccine efficacy**						
Monitoring of symptomatic COVID-19 cases (ex-program visit) [3]	●					
Blood sampling	●			●		
**Safety evaluation**						
Handover of diary form to volunteers [4]	●		●			

Returning and reviewing of Diary form			●	●		
Recording adverse event	●					
Recording AEs which need medical intervention and those end up with study stop Recording those interventions (treatments) for AEs	●					
Recording SAEs and their related treatments	●					
Recording other treatments and vaccines during the study period [5]	●					

§ Among cities under the two-dose scheme (Cohort), just Sari and Babol will do serologic assays for all volunteers on days 0 and 56 and 30% of subjects on day 5 and another 30% on day 28.

1) Window period means an acceptable interval that a volunteer can come for a visit or to receive the intervention

2) A comprehensive physical examination encompasses measuring vital signs, height and weight on day 0. Before every vaccine shot, the volunteer's arm is inspected for regional lymph nodes. Every significant physical finding must be reported as a side event that needs medical intervention. Vital signs will be assessed before vaccination on days 0 and 28. Febrile subjects on day 0 (the 1st dose of vaccine) will be excluded (a temperature beyond 38°C is defined as fever). Those volunteers having fever on day 28 (the 2nd dose of vaccine) will be rescheduled for an alternative date (within the window period) to get their 2nd shot.

All volunteers will be instructed to coordinate with the call center and go to designated laboratories for a PCR test quickly within 48 hours in case of presenting with two or more of the following symptoms lasting for over 24 hours confirmed in positive PCR testing: Fever (temperature ≥38°C), chills, new cough, sore throat, nasal congestion, fatigue, muscle or body pain, headache, nausea or vomiting, diarrhea, loss of smell or taste, or at least one respiratory sign or symptom (i.e., cough, O2 saturation <91, shortness of breath, or clinical or radiographic evidence of pneumonia).

3) Nasopharyngeal and pharyngeal swabs will be taken from the symptomatic volunteer. If the volunteer cannot be present in the lab, the operator arranges a home visit/s for him/her to take samples and clinical assessment/s. If the home visit is impossible, the symptomatic volunteer is asked to make a pharyngeal gurgle with a standard method and then bring it to the lab. If PCR turns to be negative yet the symptoms last for more than 2 days, the test will be repeated after 7 days. The volunteers with positive tests who do not need hospitalization will be visited by Phone daily until full recovery of symptoms. It is important

to take note that some manifestations of COVID-19 have overlapped with systemic AEs following immunization (e.g., myalgia, headache, fever and chills). Regarding that such AEs are more common within 3 days following vaccination in case of having other symptoms of COVID-19, an infectious disease specialist judges clinically and decides whether a sample for PCR is needed. All PCR-positive cases after the 2nd shot will be counted and included to estimate vaccine efficacy (primary outcome).

4) The volunteer must record all solicited local and systemic AEs within the first 72 hours post-vaccination in their online profiles. If any volunteer did not complete the diary, an active follow-up will be done on day four. Moreover, unsolicited adverse events will be followed up 28 days after each dose, and medically attended adverse events and serious adverse events will be monitored six months after the first dose.

5) All medical treatments and other vaccines received within the 28 days after each vaccine dose as well as AEs and SAEs will be recorded. All SAEs which need medical intervention will be recorded from screening day (Day 0) till the end of follow-up (6 months after the first dose).

Table 2. Timing of activities in C2 (3-dose regimen)

No. of visit	1	2	3	4		
Objective of visit	Screening<br>Dose 1<br>Blood sampling from 2000 subjects of Zanjan city §	Dose 2	Dose 3	Blood sampling from 2000 subjects of Zanjan city §	End of monitoring efficacy	End of monitoring adverse events
Month	Month 1		Month 2	Month 3	Month 5	Month 7
Day	0	28	56	84		
Acceptable interval (open window period) [1]	0	+ 7	+ 7	+ 7		
Informed consent<br>Complete eCRF (demographic info, concurrent treatments, history of underlying conditions, etc.)	●					
Blood sampling to assess the previous infection of COVID-19 (Antibody assay)	●					
Physical examination [2]	●	●	●			
Randomization	●					
**Intervention**						
Vaccine injection and active monitoring for 0.5 hours after the shot	●	●	●			
**Evaluation of vaccine efficacy**						
Monitoring of symptomatic COVID-19 (ex-program visit) [3]	●					
Blood sampling [4]	●			●		

**Safety evaluation**						
Handover of diary form to volunteers [5]	●	●	●			
Returning and review of Diary form [5]		●	●	●		
Recording adverse event	●					
Recording AEs which need medical intervention and those end up with study stop Recording those interventions (treatments) for AEs [5]	●					
Recording SAEs and their related treatments [5]	●					
Recording other treatments and vaccines during the study period [6]	●					

§ Two cities of Zanjan and Yazd follow the three-dose scheme. Just in Zanjan city, blood samples will be taken for serologic assays from 2000 volunteers on days 0 and 84.

1) Window period means an acceptable interval that a volunteer can come for visit or to receive the intervention

2) A comprehensive physical examination encompasses the measurement of vital signs, height and weight on day 0. Before every vaccine shot, the volunteer's arm is inspected for regional lymph nodes. Every significant physical finding must be reported as a side event that needs medical intervention. Vital signs will be assessed before vaccination on days 0, 28 and 56. Febrile subjects on day 0 (the 1st dose of vaccine) will be excluded (a temperature beyond 38^{oC} is defined as fever). Those volunteers having fever on days 28 and 56 (the 2nd dose of vaccine) reschedule for an alternative date (within the window period) to get their 2nd and 3rd shots.

3) All volunteers will be instructed to coordinate with the call center and go to designated laboratories for PCR tests quickly within 48 h. Nasopharyngeal and pharyngeal swabs will be taken from the symptomatic volunteer. If the volunteer cannot be present in the lab, the operator arranges a home visit/s for him/her to take samples and clinical assessment/s. If a home visit is impossible, the symptomatic volunteer is asked to make a pharyngeal gurgle with a standard method and then bring it to the lab. If PCR turns to be negative yet the symptoms last for more than 2 days, the test will be repeated after 7 days. The volunteers with positive tests who do not need hospitalization will be visited by Phone daily until full

recovery of symptoms. It is important to take note that some manifestations of COVID-19 have overlapped with systemic AEs following immunization (e.g., myalgia, headache, fever and chills). Regarding that such AEs are more common within 3 days following vaccination in case of having other symptoms of COVID-19, an infectious disease specialist judges clinically and decides whether a sample for PCR is needed. All PCR-positive cases after the 2nd shot will be counted and included for the estimation of vaccine efficacy (primary outcome).

4) Blood samples will be taken from 2,000 volunteers in Zanjan city for serologic assay on days 0 and 84.

5) The volunteer must record all solicited local and systemic AEs within the first 72 hours post-vaccination in their online profiles. If any volunteer did not complete the diary, an active follow-up will be done on day four. Moreover, unsolicited adverse events will be followed up 28 days after each dose, and medically attended adverse events and serious adverse events will be monitored for seven months after the first dose.

6) All medical treatments and other vaccines received within the 28 days after each vaccine dose as well as AEs and SAEs will be recorded. All SAEs which need medical intervention will be recorded from screening day (Day 0) till the end of follow-up (7 months after the first dose).

Annexe 1. Acronyms

Abbreviation	Term
ACE2	Angiotensin-Converting Enzyme-2
AE	Adverse Event
AEFI	Adverse Event Following Immunization
ALT	Alanine Aminotransferase
AST	Aspartate Aminotransferase
BMI	Body Mass Index
CI	Confidence Interval
COVID-19	Coronavirus Disease 2019
COPD	Chronic Obstructive Pulmonary Disease
CRF	Case Report Form
CT	Computed Tomography
DSMB	Data and Safety Monitoring Board
DU	Dosing Unit
EC	Ethics Committee
eCRF	Electronic Case Report Form
ELISA	Enzyme-Linked Immunosorbent Assay
EU	European Union
EV	Effect Evaluation
FDA	Food and Drug Administration
FiO2	Fraction of Inspired Oxygen
GCP	Good Clinical Practice
GFR	Glomerular Filtration Rate
HR	Hazard Ratio
ICU	Intensive Care Unit
IgG	Immunoglobulin G
IgM	Immunoglobulin M
IM	Intramuscular
ITT	Intention To Treat
IV	Intravenous (ly)

LL	Lower Limit
M	Membrane Protein
MedDRA	Medical Dictionary for Regulatory Activities
MRI	Magnetic Resonance Imaging
N	SARS-CoV-2 Nucleoprotein
N/A	Not Applicable
Non-S	Non-Spike protein
PP	Per Protocol
PaO2	Partial Pressure of Oxygen, Arterial
PCR	Polymerase Chain Reaction
PI	Principal Investigator
RBD	Receptor-Binding Domain
RNA	Ribonucleic Acid
RR	Relative Risk
PCR	Polymerase Chain Reaction
S1	Spike Protein S1 Subunit
S	Spike Protein
SAE	Serious Adverse Event
SARS-CoV-2	Severe Acute Respiratory Syndrome Coronavirus 2
SD	Standard Deviation
SOP	Standard Operating Procedure
SpO2	Oxygen Saturation as Measured by Pulse Oximetry
UL	Upper Limit
VNT	Virus Neutralization Test
VE	Vaccine Efficacy
WHO	World Health Organization

Annexe 2. Information on participating cities in each city

List of participating sites in the SOBERANA trial in Iran

City	Vaccination site	Hospital	Outpatient department	Laboratory
**Zanjan**	Zanjan vaccination center, Amir Kabir Blvd, Emam street, Zanjan	Valiasr hospital, Valiasr Square, Zanjan<br>Mousavi hospital, Dr. Sobouti Blvd. Zanjan	The sixteen-hour health center, Jomhouri Eslami Blvd, Zanjan	Core facilities, Zanjan secretary of health, Amir Kabir Blvd, Emam street, Zanjan
**Hamadan**	Hamadan vaccination center, Palestine Square, Hamadan	Besat hospital, Resalat Square, Hamadan	Dibaj sixteen-hour health center, Mirzazadeh Eshghi Street, Hamadan	Farzan Molecular and Pathobiology Laboratory , The Mausoleum of Avicenna, Hamadan
**Kerman**	Kerman vaccination center, Jomhouri Eslami Blvd, Kerman	Shafa hospital, Shafa Street, Kerman	*Dr Dabiri Laboratory, Jahad Blvd,* Kerman	Iranian clinical And Surgical Pathology Laboratory, Imam Jomeh Street, Kerman
**Yazd**	Yazd vaccination center, Safaeieh, Yazd	Shahid Sadoughi hospital, Shahid Ghandi Blvd, Yazd	ImamShahr medical center,17 Shahrivar Blvd, Yazd	Haj Maghsoodi Health center, Atlasi Blvd, Yazd
**Sari**	Nasibeh University of Nursing and Midwifery, Amir Mazandarani Blvd Vesal Street, Sari	Imam Khomeini hospital, Amir Mazandarani Blvd, Sari	No. 6 Sixteen-hour health center, Saat Square, Sari	Shahid Babaei Health center, Taleghani Blvd, Salman Farsi Street, Sari
**Babol**	Babol vaccination center, Daneshagh Square, Babol	Shahid Beheshti hospital, Shahid Keshvari Square, Babol	Ali ebne Moosalreza center, Taleghani Blvd, Babol<br>Shahid Keshvari center, Navvab Street, Babol	Razi laboratory, Modarres Street, Babol
**Bandar Abbas**	Bandar Abbas vaccination center, Payambar Azam Block,<br>Bandar Abbas	Shahid Mohammadi hospital, Jomhouri Eslami Blvd, Bandar Abbas	Medical laboratory and health center in Bandar Abbas, Jomhouri Eslami Blvd, Bandar Abbas	Medical laboratory, Jomhouri Eslami Blvd, Bandar Abbas
**Isfahan**	Isfahan vaccination center, Hakim Nezami Street, Isfahan	Noor hospital, Ostandari Street, Isfahan	Navvab Safavi health center, Ahamd Abad Square, Isfahan	Bonakdar health center, Jay Street, Isfahan<br><br>Motamed health center, Taleghani Street, Isfahan

Annexe 3: Description and composition of investigational products

The initial binding of viral particles is mediated by the SARS-CoV-2 Spike (S)-glycoprotein trimer via its Receptor Binding Domain (RBD) to the host's cell surface receptor, the angiotensin-converting enzyme 2 (ACE2). By focusing on the whole S-protein or the RBD as antigen, the primary goal lies in the induction of anti-RBD antibodies interfering with the RBD-ACE2 interaction, blocking the first step of infection and usually not participating in antibody-dependent enhancement (ADE). RBD fragments in the S-glycoprotein trimer can adopt two different conformations on the virus surface: the "down" conformation with a well-camouflaged critical receptor-binding motif (RBM), and the "up" conformation with the RBM exposed and ready to bind to the ACE2 receptor in the human host cells. However, the "up" conformation also exposes the RBM epitopes to the immune system, allowing the induction of potent neutralizing antibodies. Recombinant low-molecular-weight RBD exposes the RBM and other protein epitopes that might become immunodominant, thus deflecting the immune response against less relevant epitopes in terms of neutralization. This conjugate vaccine was developed under the hypothesis that the proportion of high neutralizing antibodies would be significantly increased if the macromolecular RBD conjugate construct could mimic the RBD in the "up" conformation, thus mainly exposing the RBM surface. The RBD-recombinant sequence selected as purified antigen for the vaccine comprises amino acids 319-541 of protein S, which means a prolongation at N and C terminal extremes of the RBD structure (Thr333 to Pro527).

This recombinant RBD 319-541 includes at the C-terminal fragment an unpaired Cys538, a residue far away from the RBM and suitable for site-selective bioconjugation into a carrier protein bearing thiophilic groups such as maleimide. This biomimetic design is based on the hypothesis that by conjugating several copies of the RBD to a large carrier protein, a macromolecular construct can be obtained mimicking the 'up' RBD conformation, in which only the RBM is well exposed and available for immune recognition. Besides Cys538, the RBD contains eight additional Cys forming four disulfide bridges, three of them stabilizing the so-called RBD "core" and one within the RBM.

Conjugation technology (by Michael's addition) has been used for more than 15 years for various vaccine candidates in Finlay Institute and constitutes a well-known platform with broad evidence of performance. It is the method developed and used to produce the active ingredient of the Cuban conjugate vaccine Quimi-Hib®, which has demonstrated its safety and efficacy in the infant population and has been incorporated into the Cuban National Vaccination Program since 2004. Tetanus toxoid was selected as a carrier protein. It has a proven performance of many years in conjugate vaccines and is part of several vaccine candidates developed at the Finlay Institute. Industrial production capacities are available to manufacturing with API Quality for vaccines such as Heberpenta® and Carrier Protein Quality for conjugate vaccines such as Quimi-Hib® and pneumococcus. The immunogenic effect of Tetanus Toxoid (TT) as a carrier of viral proteins has not been assessed previously for SARS-CoV-2 or any other coronavirus. The presence of multiple T and B-cell epitopes of this highly immunogenic carrier, as part of a conjugate construction, might help potentiate cellular immunity as compared to the use of the RBD alone and even the whole S protein. In this vaccine, the CHO expression system was used to ensure proper glycosylation of amino acids 331, 343, 323 and 325 to resemble RBD's in the virus. Evidence in the literature indicates

that RBD expressed as a recombinant protein is not toxic, regardless of its exact sequence and expression system.

Composition of SOBERANA 02 vaccine: SOBERANA 02 is the first conjugated vaccine developed for SARS CoV-2 prevention, presented as opalescent white suspension that slowly tends to form a white deposit, which is easily resuspended with shaking. The antigen selected for this vaccine candidate against COVID-19 is (25µg) the recombinant receptor-binding domain (RBD) protein conjugated chemically to tetanus toxoid (TT) in a molar ratio of 6/1 (referred to RBD6-TT: conjugate with six molecules of RBD per molecule of tetanus toxoid). The storage condition for this product is 2 to 8° C.

Composition of SOBERANA Plus vaccine: The antigen selected for this vaccine candidate against COVID-19, is a dimer of RBD (50 µg) with sequence 319-541, dimerized from an interchain disulfide bridge between a cysteine at position 538 of each monomer, adsorbed on Alumina to form an Opalescent white suspension that slowly tends to form a white deposit, which is easily resuspended with shaking. Also, a single dose of SOBERANA Plus is an excellent booster of natural immunity in convalescence through a mechanism named hybrid immunity. The storage condition for this product is 2 to 8°C.

Composition of the placebo: The placebo formulation was the same as the vaccine candidates without any antigens included.

Soberana 02 Vaccine Component	Quantity in the unit of measure (0,5 mL)
RBD of SARS-CoV-2 conjugated to tetanus toxoid	25 µg
Disodium hydrogen phosphate	0.03 mg
Sodium dihydrogen phosphate	0.02 mg
Sodium Chloride	4.25 mg
H2O	0.5 ml
Aluminium hydroxide	0.5 mg

Soberana Plus Vaccine Component	Quantity in the unit of measure (0,5 mL)
RBD of SARS-CoV-2	50 µg
Disodium hydrogen phosphate	0.03 mg
Sodium dihydrogen phosphate	0.02 mg
Sodium Chloride	4.25 mg
H2O	0.5 ml
Aluminium hydroxide	1.25 mg

Placebo Component	Quantity in the unit of measure (0,5 mL)
Disodium hydrogen phosphate	0.03 mg
Sodium dihydrogen phosphate	0.02 mg
Sodium Chloride	4.25 mg
H2O	0.5 ml
Aluminium hydroxide	0.5 mg

Implementation of the Amendment

The changes detailed in this amendment will be issued as Clinical Protocol Version 2, Date 22 June 2021.

Presentation of the Amended text

The text is amended as follows:

Revised protocol heading, and page number	Original wording from clinical protocol	Revised wording from clinical protocol
2.2. inclusion criteria<br>p.8-9	o Participants with healthy conditions or/with a controlled medical condition(s) that is under control.<br>o Having Iranian nationality<br>o Residents of the cities of the study<br>~~The study will be conducted in two age subgroups in each city as follows:~~<br>~~1) Age group ≥ 65 years (10% of the studied samples: 2400 people)~~<br>~~2) 18 to 65 years old age group (90% of the studied samples: 21,600 people)~~	o Participants with healthy conditions or/with a controlled medical condition(s) that is under control.<br>o Having Iranian nationality<br>o Residents of the cities of the study
2.3 Exclusion Criteria<br>p.10	○ Tattoos on the deltoid muscle of both arms.	○ Tattoos on the deltoid muscle of both arms.<br>○ **Those in phase 1 of the national plan for establishing and expanding COVID-19 vaccination.**
Footnote<br>p.10		[1]Based on the national plan to establish and expand COVID-19 vaccination, everyone in phase 1 of vaccination priority will receive the COVID-19 vaccine in the next few weeks. These individuals are ineligible to participate in this study if they are in the following groups: All employees who provide first-line care to COVID-19 patients at high risk of contracting and transmitting the infection, including healthcare personnel in both the public and private sectors, the country's laboratory, faculty and staff of universities as well as the Ministry of Health, Treatment and Medical Education; People at higher risk of hospitalization and death due to COVID-19, including nursing home residents and nursing home staff, Veterans esp. veterans over 50% and chemical respiratory veterans living in veterans' care centers, and mentally and

		physically disabled people living in these centers.<br>A restriction applies to health workers currently in the first phase of national vaccination priorities, despite receiving general vaccinations with approved vaccines. If they wish to enrol in this trial and enter the study, there is no prohibition against it.
2.4 Criteria to Prohibit the Second/Third Dose During the Study (Exit criteria):<br>p.10	○ A diagnosis of COVID-19 with a PCR test ~~between the first dose and 14 days later~~<br>○ Severe fever (axillary temperature ≥ 38°C) for three days or allergic reaction to the previous dose of the vaccine	○ A diagnosis of COVID-19 with PCR test **<u>after the last dose of injection</u>**<br>○ Severe fever (axillary temperature ≥ 38°C) for three days or **<u>severe</u>** allergic reaction **<u>(anaphylaxis)</u>** to the previous dose of the vaccine<br>○ **<u>Receiving drugs affecting the immune system between two injections</u>**<br>○ **<u>Any disease that affects a candidate's immunity level between two injections, based on the diagnosis of a medical professional.</u>**
Added a new heading:<br>2.5 Criteria for rescheduling the vaccine (after solving the problem)		○ **<u>Having fever or acute illness within 7 days before or on the day of injection</u>**<br>○ **<u>Having high blood pressure</u>**
3.2.3 Allocation concealment<br>p.13	The orders of universally unique identifiers (UUID) are in the software and are inaccessible to the researcher.	The orders of universally unique identifiers (UUID) are in the software and are inaccessible to the researcher. **<u>Study information is collected and managed through a web system developed by the knowledge-based company "Vista Technology Ideas Group."</u>**
3.3 Reasons for unmasking UUIDs<br>p.14	Just one investigator is authorized for unmasking with the aid of the developed online platform.<br><br>The date and reason for decoding UUID will be recorded in eCRF.	<u>Just one investigator is authorized for **urgent** unmasking with the aid of the developed online platform.</u><br><br>The date and reason for decoding UUID will be recorded in eCRF. <u>Elderly people over 65 years of age, who are at high risk of contracting severe forms of the COVID-19 disease, will be proactively contacted if they are prioritized to receive the vaccine according to the national vaccination document and the announced policies of the National COVID-19 Headquarters, and will be informed of their intervention group and in the case of the placcebo group,</u>

		they will be informed about providing them access to the vaccine of the national vaccination program.<br>In the case of other groups that are prioritized to receive vaccines according to the national vaccination program, the policies announced by the National COVID-19 Headquarters and the current programs of the country, upon their request, the code will be decoded and their treatment group will be informed.<br>If the candidate has received one or two doses of the current vaccine and is prioritized for general vaccination with other vaccines, it is recommended that s/he remain in the current study and perform subsequent injections with the vaccine. However, if they want to withdraw from the study with their consent and inject another vaccine, the program administrator (sponsor) is only responsible for the vaccine received in this study and the consequences of the change of the vaccine will be their responsibility.
4.2 Immunogenicity Assessment p.15	To assess Cell-Mediated Immunity (CMI), Interferon Gamma Release Assay (IGRA) will be performed on at least ~~30 participants from Babol or Sari.~~	To assess Cell-Mediated Immunity (CMI), Interferon Gamma Release Assay (IGRA) will be performed on at least **130 participants from Babol or Sari (Control: 26; vaccine: 104).**

References

1) Chang-Monteagudo A, Ochoa-Azze R, Climent-Ruiz Y, Macías Abraham C, Rodríguez-Noda L, Valenzuela-Silva C, et al. A single dose of SARS-CoV-2 FINLAY-FR-1A dimeric-RBD recombinant vaccine enhances neutralization response in COVID-19 convalescents, with excellent safety profile. A preliminary report of an open-label phase 1 clinical trial. Doi: https://doi.org/10.1101/2021.02.22.21252091

2) world health organization. Public health surveillance for COVID-19. Interim guidance. 16 December 2020. Available at: https://www.who.int/publications/i/item/who-2019-nCoV-surveillanceguidance-2020.8

3) US Food and Drug Administration. Guidance for industry: toxicity grading scale for healthy adult and adolescent volunteers enrolled in preventive vaccine clinical trials. Rockville, MD: Center for Biologics Evaluation and Research; September 2007.

4) Li F. Structure, Function, and Evolution of Coronavirus Spike Proteins. Annu Rev Virol. 02016;3(1):237-261.

5) World Health Organization. WHO Coronavirus Disease (COVID-19) Dashboard. 2020; .2https://COVID19.who.int/. Accessed Sep 3, 2020.

6) Yu F, Du L, Ojcius DM, Pan C, Jiang S. Measures for diagnosing and treating infections .2 by a novel coronavirus responsible for a pneumonia outbreak originating in Wuhan, China. Microbes and Infection. 2020;22(2):74-79.

7) Lu R, Zhao X, Li J, et al. Genomic characterisation and epidemiology of 2019 novel .2 coronavirus: implications for virus origins and receptor binding. The Lancet. 2020;395(10224):565-574.

8) Li Q, Guan X, Wu P, et al. Early Transmission Dynamics in Wuhan, China, of Novel .22 Coronavirus-Infected Pneumonia. N Engl J Med. 2020;382(13):1199-1207. Wrapp D, Wang N, Corbett KS, et al. Cryo-9) EM structure of the 2019-nCoV spike in the .22 prefusion conformation. Science (New York, NY). 2020;367(6483):1260-1263.

9) Magalhães, N., Oliveira, H.M., & Alves, A.J. (2008). On preparing a list of random treatment assignments.

10) M. Matsumoto and T. Nishimura, “Mersenne Twister: A 623-dimensionally equidistributed uniform pseudorandom number generator”, ACM Transactions on Modeling and Computer Simulation Vol. 8, No. 1, January pp.3–30 1998
