# Supplementary material for: Efficacy and Safety of a Protein-Based SARS-CoV-2 Vaccine: A Randomized Clinical Trial
Source: JAMA Netw Open. 2023 May 3;6(5):e2310302. doi: 10.1001/jamanetworkopen.2023.10302 (PMC10157429; doi:10.1001/jamanetworkopen.2023.10302)
Supplement: Supplement 2. — eFigure 1. Study Sites of the SOBERANA trial in Iran eAppendix. List of Participating Sites and Address of Different Units Within Each City eMethods. Detailed Methods eFigure 2. Schoenfeld Residual Plots and Estimated Log-Log Survival Curves for Assessing the Proportional Hazard Assumption eTable 1. Vaccine Efficacy Against SARS-CoV-2 14 Days in Different Subgroups Based on Stratified Cox Regression eTable 2. Vaccine Efficacy Against SARS-CoV-2 Using the Logistic Model eTable 3. Vaccine Efficacy Against SARS-CoV-2 in Different Subgroups Based on Logistic Regression eResults. Detailed Results eFigure 3. Humoral Immune Response (Anti SARS-CoV-2 S1 IgG and Neutralizing Antibodies Titers) in the Vaccine and Placebo Groups eTable 4. Seroconversion Rate in Different Subgroups After the First, Second, and Third Doses eTable 5. Adverse Events (AEs) From Day 0 to Day 28 eTable 6. Local and Systemic Adverse Events After Injection of the First, Second and Third Doses eTable 7. Serious and Medically Attended Adverse Events (SAEs) From Day 0 Up to Day 120 eTable 8. Serious and Medically Attended Adverse Events by MedDRA System Organ Class During the Study, in Randomized Participants Who Received at Least One Dose eTable 9. The Number Needed to Vaccinate in Cohort 1 and Cohort 2 eTable 10. Reason for Unmet Inclusion Criteria in Screened Participants eFigure 4. Percentage of Different SARS-CoV-2 Variants of Concern During the Study eReferences [file jamanetwopen-e2310302-s002.pdf]

Supplementary Online Content

Mostafavi E, Eybpoosh S, Karamouzian M, et al. Efficacy and safety of a protein-based SARS-CoV-2 vaccine: a randomized clinical trial. *JAMA Netw Open*. 2023;6(5):e2310302. doi:10.1001/jamanetworkopen.2023.10302

eFigure 1. Study Sites of the Soberana Trial in Iran

eAppendix. List of Participating Sites and Address of Different Units Within Each City

eMethods. Detailed Methods

eFigure 2. Schoenfeld Residual Plots and Estimated Log-Log Survival Curves for Assessing the Proportional Hazard Assumption

eTable 1. Vaccine Efficacy Against SARS-CoV-2 14 Days in Different Subgroups Based on Stratified Cox Regression

eTable 2. Vaccine Efficacy Against SARS-CoV-2 Using the Logistic Model

eTable 3. Vaccine Efficacy Against SARS-CoV-2 in Different Subgroups Based on Logistic Regression

eResults. Detailed Results

eFigure 3. Humoral Immune Response (Anti SARS-CoV-2 S1 IgG and Neutralizing Antibodies Titers) in the Vaccine and Placebo Groups

eTable 4. Seroconversion Rate in Different Subgroups After the First, Second, and Third Doses

eTable 5. Adverse Events (AEs) From Day 0 to Day 28

eTable 6. Local and Systemic Adverse Events After Injection of the First, Second and Third Doses

eTable 7. Serious and Medically Attended Adverse Events (SAEs) From Day 0 Up to Day 120

eTable 8. Serious and Medically Attended Adverse Events by MedDRA System Organ Class During the Study, in Randomized Participants Who Received at Least One Dose

eTable 9. The Number Needed to Vaccinate in Cohort 1 and Cohort 2

eTable 10. Reason for Unmet Inclusion Criteria in Screened Participants

eFigure 4. Percentage of Different SARS-CoV-2 Variants of Concern During the Study

eReferences

This supplementary material has been provided by the authors to give readers additional information about their work.

© 2023 Mostafavi E et al. *JAMA Network Open.*

eFigure 1. Study sites of the SOBERANA trial in Iran. The sample size in each city was 3000 people.

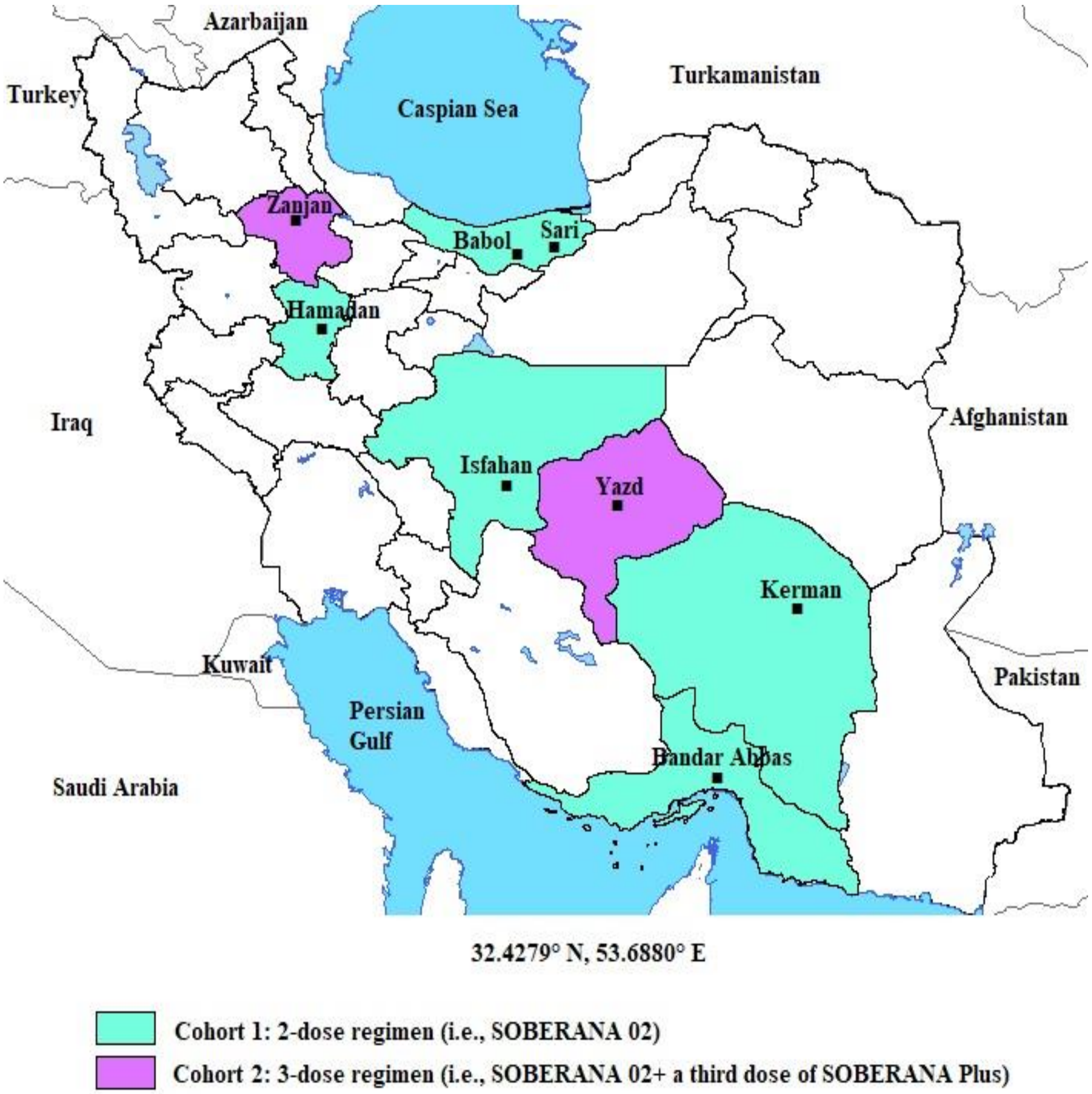

© 2023 Mostafavi E et al. *JAMA Network Open.*

eAppendix. List of participating sites and address of different units within each city

City	Vaccination site	Hospital	Outpatient department	Laboratory
**Zanjan**	Zanjan vaccination center, Amir Kabir Blvd, Emam street, Zanjan	Valiasr hospital, Valiasr Square, Zanjan<br>Mousavi hospital, Dr. Sobouti Blvd. Zanjan	The sixteen-hour health center, Jomhouri Eslami Blvd, Zanjan	Core facilities, Zanjan secretary of health, Amir Kabir Blvd, Emam street, Zanjan
**Hamadan**	Hamadan vaccination center, Palestine Square, Hamadan	Besat hospital, Resalat Square, Hamadan	Dibaj sixteen-hour health center, Mirzazadeh Eshghi Street, Hamadan	Farzan Molecular and Pathobiology Lab oratory, The Mausoleum of Avicenna, Hamadan
**Kerman**	Kerman vaccination center, Jomhouri Eslami Blvd, Kerman	Shafa hospital, Shafa Street, Kerman	Dr. Dabiri Laboratory, Jahad Blvd, Kerman	Iranian clinical And Surgical Pathology Laboratory, Imam Jomeh Street, Kerman
**Yazd**	Yazd vaccination center, Safaeieh, Yazd	Shahid Sadoughi hospital, Shahid Ghandi Blvd, Yazd	ImamShahr medical center,17 Shahrivar Blvd, Yazd	Haj Maghsoodi Health center, Atlasi Blvd, Yazd
**Sari**	Nasibeh University of Nursing and Midwifery, Amir Mazandarani Blvd Vesal Street, Sari	Imam Khomeini hospital, Amir Mazandarani Blvd, Sari	No. 6 Sixteen-hour health center, Saat Square, Sari	Shahid Babaei Health center, Taleghani Blvd, Salman Farsi Street, Sari
**Babol**	Babol vaccination center, Daneshagh Square, Babol	Shahid Beheshti hospital, Shahid Keshvari Square, Babol	Ali ebne Moosalreza center, Taleghani Blvd, Babol<br><br>Shahid Keshvari center, Navvab Street, Babol	Razi laboratory, Modarres Street, Babol
**Bandar Abbas**	Bandar Abbas vaccination center, Payambar Azam Block, Bandar Abbas	Shahid Mohammadi hospital, Jomhouri Eslami Blvd, Bandar Abbas	Medical laboratory and health center in Bandar Abbas, Jomhouri Eslami Blvd, Bandar Abbas	Medical laboratory, Jomhouri Eslami Blvd, Bandar Abbas
**Isfahan**	Isfahan vaccination center, Hakim Nezami Street, Isfahan	Noor hospital, Ostandari Street, Isfahan	Navvab Safavi health center, Ahamd Abad Square, Isfahan	Bonakdar health center, Jay Street, Isfahan<br><br>Motamed health center, Taleghani Street, Isfahan

© 2023 Mostafavi E et al. *JAMA Network Open.*

eMethods. Detailed Methods

2.1 Description and composition of investigational products

The initial binding of viral particles is mediated by the SARS-CoV-2 Spike (S)-glycoprotein trimer via its Receptor Binding Domain (RBD) to the host's cell surface receptor, the angiotensin-converting enzyme 2 (ACE2). By focusing on the whole S-protein or the RBD as antigen, the primary goal lies in the induction of anti-RBD antibodies interfering with the RBD-ACE2 interaction, blocking the first step of infection and usually not participating in antibody-dependent enhancement (ADE). RBD fragments in the S-glycoprotein trimer can adopt two different conformations on the virus surface: the "down" conformation with a well-camouflaged critical receptor-binding motif (RBM), and the "up" conformation with the RBM exposed and ready to bind to the ACE2 receptor in the human host cells. However, the "up" conformation also exposes the RBM epitopes to the immune system, allowing the induction of potent neutralizing antibodies. Recombinant low-molecular weight RBD exposes not only the RBM but also other protein epitopes that might become immunodominant, thus deflecting the immune response against less relevant epitopes in terms of neutralization. This conjugate vaccine was developed under the hypothesis that the proportion of high neutralizing antibodies would be significantly increased if the macromolecular RBD conjugate construct could mimic the RBD in the "up" conformation, thus mainly exposing the RBM surface. The RBD-recombinant sequence selected as purified antigen for the vaccine comprises amino acids 319-541 of protein S, which means a prolongation at N and C terminal extremes of the RBD structure (Thr333 to Pro527).
This recombinant RBD 319-541 includes at the C-terminal fragment an unpaired Cys538, a residue far away from the RBM and suitable for site-selective bioconjugation into a carrier protein bearing thiophilic groups such as maleimide. This biomimetic design is based on the hypothesis that by conjugating several copies of the RBD to a large carrier protein, a macromolecular construct can be obtained mimicking the 'up' RBD conformation, in which only the RBM is well exposed and available for immune recognition. Besides Cys538, the RBD contains eight additional Cys forming four disulfide bridges, three of them stabilizing the so-called RBD "core" and one within the RBM.
Conjugation technology (by Michael's addition) has been used for more than 15 years for various vaccine candidates in Finlay Institute and constitutes a well-known platform with broad evidence of performance. It is the method developed and used to produce the active ingredient of the Cuban conjugate vaccine Quimi-Hib®, which has demonstrated its safety and efficacy in the infant population and has been incorporated into the Cuban National Vaccination Program since 2004. Tetanus toxoid was selected as a carrier protein. It has a proven performance of many years in conjugate vaccines and is part of several vaccine candidates developed at the Finlay institute. Industrial production capacities are available to manufacturing with API Quality for vaccines such as Heberpenta® and Carrier Protein Quality for conjugate vaccines such as Quimi-Hib® and pneumococcus. The immunogenic effect of Tetanus Toxoid (TT) as a carrier of viral proteins has not been assessed previously for SARS-CoV-2 or any other coronavirus. The presence of multiple T and B-cell epitopes of this highly immunogenic carrier, as part of a conjugate construction, might help potentiate cellular immunity as compared to the use of the RBD alone and even the whole S protein. In this vaccine, the CHO expression system was used to ensure proper glycosylation of amino acids 331, 343, 323 and 325 to resemble that of RBD in the virus. Evidence in the literature indicates that RBD expressed as a recombinant protein is not toxic, regardless of its exact sequence and expression system.
SOBERANA 02 and SOBERANA Plus vaccines are developed and produced at the Finlay Vaccine Institute of Cuba and manufactured at Pasteur institute of Iran as Pastocovac® and Pastocovac plus® after a successful technology transfer.

Composition of SOBERANA 02 vaccine: SOBERANA 02 is the first conjugated vaccine developed for SARS CoV-2 prevention, presented as an opalescent white suspension that slowly tends to form a white deposit, which is easily re-suspended with shaking. The antigen selected for this vaccine candidate against COVID-19 is (25µg) the recombinant receptor binding domain (RBD) protein conjugated chemically to tetanus toxoid (TT) in a molar ratio of 6/1 (referred to RBD6-TT: conjugate with six molecules of RBD per molecule of tetanus toxoid). The storage condition for this product is 2 to 8° C.

Composition of SOBERANA Plus vaccine: The antigen selected for this vaccine candidate against COVID-19, is a dimer of RBD (50 µg) with sequence 319-541, dimerized from an interchain disulfide bridge between a cysteine at position 538 of each monomer, adsorbed on Alumina to form an Opalescent white suspension that slowly tends to form a white deposit, which is easily resuspended with shaking. Also, a single dose of SOBERANA Plus is an

excellent booster of natural immunity in convalescence through a mechanism named hybrid immunity. The storage condition for this product is 2 to 8°C.

Composition of the placebo: The placebo formulation was the same as the vaccine candidates without any antigens included.

Soberana 02 Vaccine Component	Quantity in the unit of measure (0,5 mL)
RBD of SARS-CoV-2 conjugated to tetanus toxoid	25 µg
Disodium hydrogen phosphate	0.03 mg
Sodium dihydrogen phosphate	0.02 mg
Sodium Chloride	4.25 mg
H2O	0.5 ml
Aluminum hydroxide	0.5 mg

Soberana Plus Vaccine Component	Quantity in the unit of measure (0,5 mL)
RBD of SARS-CoV-2	50 µg
Disodium hydrogen phosphate	0.03 mg
Sodium dihydrogen phosphate	0.02 mg
Sodium Chloride	4.25 mg
H2O	0.5 ml
Aluminum hydroxide	1.25 mg

Placebo Component	Quantity in the unit of measure (0,5 mL)
Disodium hydrogen phosphate	0.03 mg
Sodium dihydrogen phosphate	0.02 mg
Sodium Chloride	4.25 mg
H2O	0.5 ml
Aluminum hydroxide	0.5 mg

© 2023 Mostafavi E et al. *JAMA Network Open.*

2.2 Endpoint Definitions of Covid-19 cases

Symptomatic cases were defined as those presenting with two or more of the following symptoms lasting for over 24 hours confirmed with an additional positive RT-PCR testing: Fever (temperature ≥38°C), chills, sore throat, nasal congestion, fatigue, muscle or body pain, headache, nausea or vomiting, diarrhoea, loss of smell or taste, or at least one respiratory sign or symptom (i.e., cough, shortness of breath, or clinical or radiographic evidence of pneumonia).

Severe cases were defined as patients with O2 saturation level ≤90%, evidence of lower respiratory disease (e.g., shortness of breath, chest pain, or chest tightness) with or without fever ≥38°C during clinical assessment or imaging, tachypnea (i.e., respiration rate >30 breaths/min), increased P(A-a) O2 gradient, lung infiltration >50% in CT-scans, PaO2/FiO2 <300 mmHg, or acute worsening of respiratory symptoms especially dyspnea, respiratory failure, septic shock, and/or multiple organ dysfunction, hospitalization or death due to COVID-19.

© 2023 Mostafavi E et al. *JAMA Network Open.*

2.3 Global Initiative on Sharing Avian Influenza Data (GISAID) accession numbers

EPI_ISL_5885499-602, EPI_ISL_5885604-98, EPI_ISL_5885702-6, EPI_ISL_5885708-70, EPI_ISL_5885772, EPI_ISL_5885774, EPI_ISL_5885777-86, EPI_ISL_5885789, EPI_ISL_5885793-9, EPI_ISL_5885804, EPI_ISL_5885808 to EPI_ISL_5885811, EPI_ISL_5885814, EPI_ISL_5885817-18, EPI_ISL_5885821-23, EPI_ISL_5885828-32, EPI_ISL_5885835, EPI_ISL_5885837, EPI_ISL_5885839-48, EPI_ISL_5885850, EPI_ISL_5885852-62, EPI_ISL_5885865, EPI_ISL_5885867-75, EPI_ISL_5885879, EPI_ISL_5885885, EPI_ISL_5885889, EPI_ISL_5885894, EPI_ISL_5885898, EPI_ISL_5885903-6, EPI_ISL_5885908, EPI_ISL_5885914-7, EPI_ISL_5885922, EPI_ISL_5885928, EPI_ISL_5885933, EPI_ISL_5885939, EPI_ISL_5885944, EPI_ISL_5885950-2, EPI_ISL_5885958-91, EPI_ISL_5885996, and EPI_ISL_6134610 -26.

© 2023 Mostafavi E et al. *JAMA Network Open.*

2.4 Sample size calculation

Separate power calculations were performed for the two cohorts. To demonstrate a clinically meaningful difference, we assumed 50% efficacy for two-dose regimens (C1) based on WHO criteria and hypothesized 70% efficacy for the three-dose regimen (C2) based on an independent Technical Support Committee (TSC) opinion that considered the immune response results observed in phases I and II trials of the 2 and 3 dose regimens[1-4] (1-4). Other assumptions included an exponential distribution, minimum baseline incidence of disease in two (0.012) and three-month (0.018) follow-ups (incidence rate: 0.0055 per month)[5,6], maximum dropout proportion of 10% and 20%[7,8], and a vaccine to placebo ratio of 4:1. Type I (a two-sided hypothesis) and type II errors of 0.05 and 0.1 were considered for sample size calculation in each cohort. The total sample size was calculated to be about 18,000 participants in C1 (14,400 in the vaccine and 3,600 in the placebo group) and 6,000 participants in C2 (4,800 in the vaccine and 1,200 in the placebo group) using Stata software.

Software command for sample size calculation
Software: Stata (v.14.2)

Cohort 1
power exponential 0.0055, hratio(0.5) power(0.9) nratio(4) fperiod(2) lossprob(0.1) losstime(2)

Cohort 2
power exponential 0.0055, hratio(0.3) power(0.9) nratio(4) fperiod(3) lossprob(0.2) losstime(3)

© 2023 Mostafavi E et al. *JAMA Network Open.*

2.5 Sampling Procedure of the Serology Subset

An anti-SARS-CoV-2 ELISA kit was used to determine anti-S1 IgG antibodies in serum samples of a subgroup of the studied population.

We selected two of the six cities (Sari and Babol) in C1 and one of the two cities (Zanjan) in C2 using a pseudo-random number generator. In the selected cities of Sari and Babol, the blood samples of all participants were tested, and in Zanjan, participants were selected via simple random sampling. Serum samples were obtained from ~30% of all the participants (C1: n=5,905 in Sari and Babol) and (C2: n=1,975 in Zanjan) on day 0.

© 2023 Mostafavi E et al. *JAMA Network Open.*

2.6 Statistical analysis

Follow-up time was divided into fixed one-week intervals to get a discrete-time survival (long person-time) format. VE along was calculated using a stratified cox proportional hazard (PH) regression model, where the cities were included in the model as the stratum variable. The PH assumption was assessed using Schoenfeld residuals for the stratified Cox proportional regression model and log-log plot (eFigure 2). A sensitivity analysis was conducted to evaluate the extreme scenarios of complete positive and negative correlations between censoring and events of interest to ensure the independent censoring assumption was met. As a sensitivity analysis, VE was also estimated through a logistic regression on the long person-time format dataset using an exchangeable correlation matrix (eTables 2 and 3). VE was defined as a 1 – exp(β). In both models, exp(β) could be a reasonable estimation of the risk ratio (RR).[9-11] Standard errors were calculated using a robust estimation method. The origin and the start of the follow-up time were the exact dates as randomization. The data lock date was August 22, 2021, for C1 and October 02, 2021, for C2.

The effects of age, sex, and underlying diseases on the VE were assessed in bivariable models. Cumulative incidence curves (1 – Kaplan-Meier estimate) were plotted. The number needed to vaccinate (NNV) was calculated.

© 2023 Mostafavi E et al. *JAMA Network Open.*

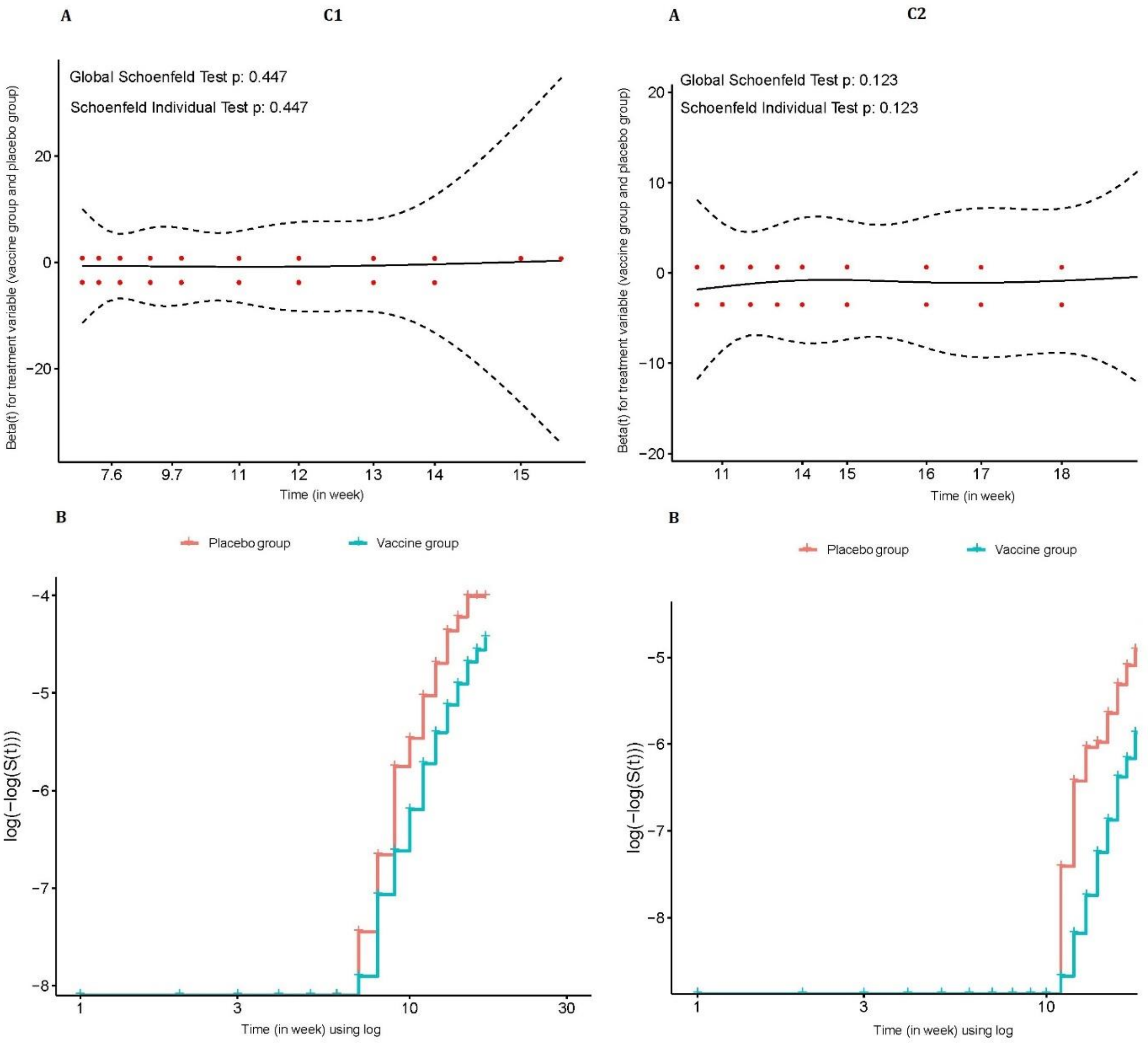

eFigure 2. Schoenfeld residual plots and estimated log-log survival curves for assessing the proportional hazard assumption.

Notes: A: Schoenfeld residual plots and B: log-log survival curves. The estimated log-log survival curves and Schoenfeld residual plots for the primary outcome show the PH assumption is satisfied in both C1 and C2.

© 2023 Mostafavi E et al. *JAMA Network Open.*

eTable 1. Vaccine Efficacy against SARS-CoV-2 14 days in different subgroups based on stratified cox regression

	Cohort 1 (C1)				Cohort 2 (C2)			
**Symptomatic COVID-19 cases**								
**Variables**	Placebo	Vaccine	VE (95% CI*)	*P*[*]	Placebo	Vaccine	VE (95% CI*)	*P*[*]
**Gender**								
Men	124/2129 (5.8%)	290/8648 (3.4%)	44.1% (30.8-54.9)	0.120	32/717 (4.5%)	47/2869 (1.6%)	64.9% (44.7-77.7)	0.983
Women	97/1471(6.6%)	171/5752 (3.0%)	57.2% (44.9-66.8)		19/483 (3.9%)	28/1931 (1.5%)	64.4% (35.7-80.3)	
**Age**								
≤65	219/3514 (6.2%)	458/14055 (3.3%)	49.6% (40.7-57.2)	0.730	51/1172 (4.4%)	75/4683 (1.6%)	64.9% (49.7-75.5)	NA
>65	2/86 (2.3%)	3/345 (0.9%)	51.3% (-189.4-91.8)[¥]		0/28 (0.0%)	0/117 (0.0%)	NA	
**Underlying disease**								
Yes	56/973 (5.8%)	151/4266 (3.5%)	40.6% (18.9-56.5)	0.200	15/352 (4.3%)	23/1448 (1.6%)	63.3% (29.1-81.1)	0.890
No	165/2627 (6.3%)	310/10134 (3.0%)	53.2% (43.3-61.3)		36/848 (4.2%)	52/3352 (1.6%)	65.4% (46.9-77.5)	
**Severe COVID-19 cases**								
**Gender**								
Men	14/2129 (0.7%)	22/8648 (0.3%)	62.4% (26.2-80.8)	0.036	4/717 (0.6%)	1/2869 (0.0%)	94.1% (47.4-99.3)	NA
Women	11/1471 (0.7%)	4/5752 (0.1%)	90.9% (70.8-97.1)		3/483 (0.6%)	0/1931 (0.0%)	NA	
**Age**								
≤65	25/3514(0.7%)	26/14055 (0.2%)	74.9% (56.4-85.5)	NA	7/1172 (0.6%)	1/4682 (0.0%)	96.6% (72.2-99.6)	NA
>65	0/86 (0.0%)	0/345 (0.0%)	NA		0/28 (0.0%)	0/117 (0.0%)	NA	
**Underlying disease**								
Yes	13/973 (1.3%)	12/4266 (0.3%)	79.5% (55.0-90.7)	0.540	2/352 (0.6%)	1/1448 (0.1%)	88.2% (-30.2-98.9)[¥]	NA
No	12/2627 (0.5%)	14/10134 (0.1%)	71.1% (37.3-86.7)		5/848 (0.6%)	0/3352 (0.0%)	NA	

Notes: NA: Not applicable due to small cell sizes.
VE: Vaccine Efficacy; CI: Confidence Interval
¥The VE estimate is unstable due to the relatively small number of participants and the low number of observed events, resulting in a very wide confidence interval.
*P-values were obtained based on the significance of interaction terms.

© 2023 Mostafavi E et al. *JAMA Network Open.*

eTable 2. Vaccine efficacy against SARS-CoV-2 using the logistic model

Condition	Total cases	Placebo	Vaccine	Vaccine efficacy (95%CI)
**14 days after the second dose**				
Confirmed symptomatic COVID-19	682	221/3600 (6.1%)	461/14400 (3.2%)	49.4% (40.5-56.9)
Severe cases	51	25/3600 (0.7%)	26/14400 (0.2%)	74.6% (56.0-85.3)
Hospitalization	48	25/3600 (0.7%)	23/14400 (0.2%)	77.5% (60.2-87.2)
**14 days after the third dose**				
Confirmed symptomatic COVID-19	126	51/1200 (4.3%)	75/4800 (1.6%)	64.6% (49.4-75.2)
Severe cases	8	7/1200 (0.6%)	1/4800 (0.0%)	96.6% (72.0-99.6)
Hospitalization	8	7/1200 (0.6%)	1/4800 (0.0%)	96.6% (72.0-99.6)

© 2023 Mostafavi E et al. *JAMA Network Open.*

eTable 3 Vaccine efficacy against SARS-CoV-2 in different subgroups based on logistic regression

	Cohort 1 (C1)				Cohort 2 (C2)			
**Symptomatic COVID-19 cases**								
**Variables**	Placebo	Vaccine	VE (95% CI*)	*P*[*]	Placebo	Vaccine	VE (95% CI*)	*P*[*]
**Gender**								
Men	124/2129 (5.8%)	290/8648 (3.4%)	43.8% (30.5-54.5)	0.121	32/717 (4.5%)	47/2869 (1.6%)	64.7% (44.5-77.5)	0.977
Women	97/1471(6.6%)	171/5752 (3.0%)	56.8% (44.4-66.4)		19/483 (3.9%)	28/1931 (1.5%)	64.1% (35.2-80.1)	
**Age**								
≤65	219/3514 (6.2%)	458/14055 (3.3%)	49.3% (40.4-56.9)	0.885	51/1172 (4.4%)	75/4683 (1.6%)	64.6% (49.4-75.2)	NA
>65	2/86 (2.3%)	3/345 (0.9%)	49.2% (-221.9-92.0)[¥]		0/28 (0.0%)	0/117 (0.0%)	NA	
**Underlying disease**								
Yes	56/973 (5.8%)	151/4266 (3.5%)	40.1% (18.4-56.1)	0.198	15/352 (4.3%)	23/1448 (1.6%)	63.2% (29.2-80.9)	0.910
No	165/2627 (6.3%)	310/10134 (3.0%)	52.8% (42.9-61.0)		36/848 (4.2%)	52/3352 (1.6%)	65.0% (46.4-77.1)	
**Severe COVID-19 cases**								
**Gender**								
Men	14/2129 (0.7%)	22/8648 (0.3%)	62.0% (25.6-80.6)	0.036	4/717 (0.6%)	1/2869 (0.0%)	94.0% (46.9-99.3)	NA
Women	11/1471 (0.7%)	4/5752 (0.1%)	90.8% (70.5-97.1)		3/483 (0.6%)	0/1931 (0.0%)	NA	
**Age**								
≤65	25/3514(0.7%)	26/14055 (0.2%)	74.6% (56.0-85.4)	NA	7/1172 (0.6%)	1/4682 (0.0%)	96.6% (72.0-99.6)	NA
>65	0/86 (0.0%)	0/345 (0.0%)	NA		0/28 (0.0%)	0/117 (0.0%)	NA	
**Underlying disease**								
Yes	13/973 (1.3%)	12/4266 (0.3%)	79.4% (54.6-90.6)	0.538	2/352 (0.6%)	1/1448 (0.1%)	88.1% (-29.4-98.9)[¥]	NA
No	12/2627 (0.5%)	14/10134 (0.1%)	70.8% (36.5-86.5)		5/848 (0.6%)	0/3352 (0.0%)	NA	

Notes: NA: Not applicable due to small cell sizes.
VE: Vaccine Efficacy; CI: Confidence Interval
¥The VE estimate is unstable due to the relatively small number of participants and the low number of observed events, resulting in a very wide confidence interval.
*P-values were obtained based on the significance of interaction terms.

© 2023 Mostafavi E et al. *JAMA Network Open.*

eResults. Detailed Results

2.7 Kinetics of SARS-CoV-2 anti-S IgG and neutralizing antibodies after vaccination

The geometric mean titer (GMT) of IgG in the vaccine group four weeks after the first, second, and third doses were 11.1 RU/ml [9.9-12.4], 119.2 RU/ml [114.1-124.5], and 133.8 RU/ml [128.9-138.8], respectively (eFigure 3). The GMT ratio between the titers after the second and first dose was 10.7 [9.5-12.2] ($P<0.001$), and between the third and second doses was 1.1 [1.06-1.2] ($P<0.001$). The corresponding GMT four weeks after the first, second, and third doses were 1.4 RU/ml [1.2-1.7], 4.5 RU/ml [3.9-5.1], and 3.3 RU/ml [2.6-3.8] in the placebo group. In the vaccine group, the seroconversion rates increased four weeks after the second (81.1% [79.7-82.2]) and third (92.9% [91.4-94.2]) doses ($P<0.001$). In the placebo group, the seroconversion rate was 5.6% ([4.2-7.3]; $P < 0.001$ vs. the vaccine group) after the second dose, and 3.0% ([1.4-5.5]; $P < 0.001$ vs. the vaccine group) after the third dose (eTable 4).

To evaluate the induction of neutralizing antibodies, random serum samples from 112 participants (C1: 11 from the placebo group, 43 from the vaccine group, and C2: 6 from the placebo group, 52 from the vaccine group) were tested (eFigure 3). In the vaccine group, the GMT of neutralizing antibodies in C1 samples on day 56 was 44.9 (28.5, 70.3), and the seroconversion rate was 69.8% (53.8, 82.8). The GMT of neutralizing antibodies in C2 samples from the vaccine group on day 84 was 133 (89.8, 178.4), and the seroconversion rate was 100% (eFigure 3). All samples from the placebo group tested negative for neutralizing antibodies (GMT = 0 and seroconversion rate = 0; $P = 0.002$ vs. the vaccine group).

© 2023 Mostafavi E et al. *JAMA Network Open.*

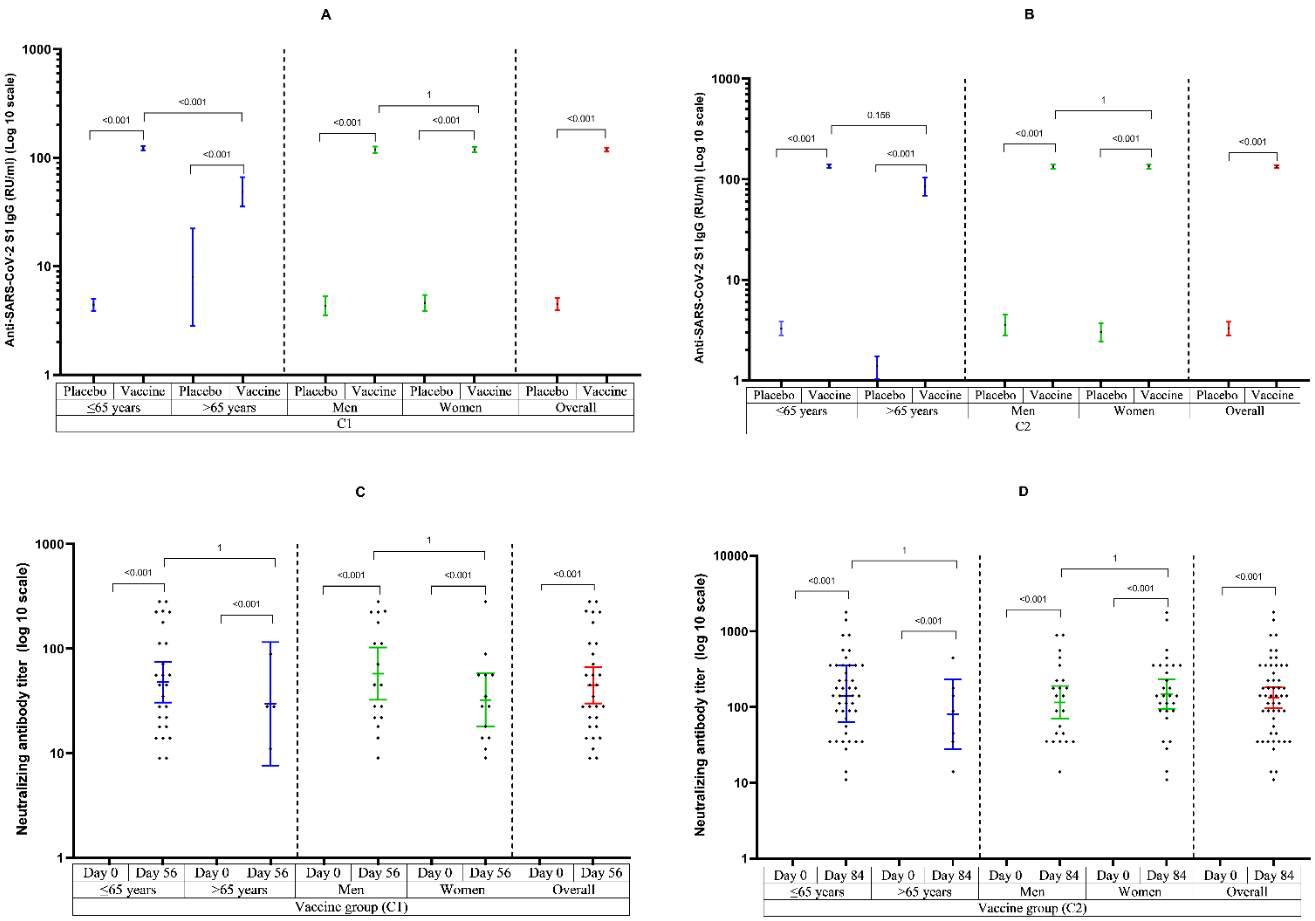

eFigure 3: Humoral immune response (Anti SARS-CoV-2 S1 IgG and neutralizing antibodies titers) in the vaccine and placebo groups.

Notes: A) Anti-SARS-CoV-2 S1 IgG titer on the logarithmic scale among participants who received the vaccine by sex and age group and also overall vaccine and placebo on day 56 (C1). B) Anti-SARS-CoV-2 S1 IgG on the logarithmic scale titer among participants who received the vaccine by sex and age group and also overall vaccine and placebo on day 84 (C2). C) Neutralizing antibodies titer on the logarithmic scale among participants who received the vaccine in C1. D) Neutralizing antibodies titer on the logarithmic scale among participants who received the vaccine in C2. The values of neutralizing antibodies for all placebo samples were not shown in Panels C and D (all were negative (titer = 0)). Error bars show geometric mean titers with 95% CI, dots show individual data points. P-values were calculated using the nonparametric bootstrap method with 1000 repetitions and have been adjusted by the Bonferroni correction for multiple comparisons; $min\{1, m\times P\text{-}value\}$ where m indicates the number of comparisons.

© 2023 Mostafavi E et al. *JAMA Network Open.*

eTable 4. Seroconversion rate in different subgroups after the first, second, and third doses

	Day 28			Day 56 (C1)			Day 84 (C2)		
**Variables**	**Vaccine**	**Placebo**	***P***	**Vaccine**	**Placebo**	***P***	**Vaccine**	**Placebo**	***P***
**Overall**	39.3% (36.7-42)	5.8% (3.4-9.1)	<0.001	81.1% (79.7-82.2)	5.6% (4.2-7.3)	<0.001	92.9% (91.4-94.2)	3% (1.4-5.5)	<0.001
**Gender**									
Men	39.5% (36.3-43.2)	8.5% (4.8-13.6)	<0.001	82.2% (80.5-83.8)	6.1% (4.2-8.4)	<0.001	94.1% (92.1-95.6)	2.3% (0.6-5.7)	<0.001
Women	38.8% (34.7-42.9)	2.3% (0.4-6.4)	<0.001	79.6% (77.5-81.4)	5% (3.1-7.5)	<0.001	91.5% (89-93.5)	3.9% (1.4-8.2)	<0.001
**P-value**	0.794	0.021		0.040	0.466		0.054	0.400	
**Age**									
≤65	39.9% (37.2-42.6)	5.8% (3.5-9.1)	<0.001	81.4% (80.1-82.6)	5.5% (4.1-7.2)	<0.001	92.9% (91.3-94.2)	3% (1.4-5.5)	<0.001
>65	22.7% (11.4-37.8)	0%	<0.001	69.6% (60.2-77.8)	10.5% (1.3-33.1)	<0.001	93.9% (79.7-99.3)	0%	<0.001
**P-value**	0.021	0.665		0.001	0.348		0.599	0.860	
**Underlying diseases**									
Yes	42.6% (38.1-47.2)	9% (3.9-16.9)	<0.001	78.9% (76.7-81.1)	6% (3.6-9.2)	<0.001	91% (87.4-93.8)	2.7% (0.3-9.3)	<0.001
No	37.6% (34.4-40.8)	4.5% (2.2-8.2)	<0.001	82.3% (80.7-83.8)	5.5% (3.8-7.5)	<0.001	93.6% (91.9-95)	3.2% (1.4-6.1)	<0.001
**P-value**	0.071	0.125		0.009	0.753		0.103	0.825	
**Anti-SARS-CoV-2 S1 IgG on Day 0**									
Positive	50% (21.1-78.9)	0%	0.049	69.7% (67.1-72.2)	1.2% (0.3-3.1)	<0.001	76.7% (72-80.9)	0%	<0.001
Negative	39.3% (36.6-41.9)	5.9% (3.5-9.2)	<0.001	86.5% (85.2-87.8)	8% (5.9-10.3)	<0.001	98.8% (97.9-99.4)	4% (1.9-7.3)	<0.001
**P-value**	0.450	0.575		<0.001	<0.001		<0.001	0.071	

© 2023 Mostafavi E et al. *JAMA Network Open.*

2.8 Vaccine efficacy estimates among the participants with a serological assessment at the baseline

Of participants who underwent a serological test on day 0 (C1= 5,905, C2= 1,975), 35.7% and 33.7% of those in the vaccine groups, as well as 36.7% and 29.7% in the placebo groups had anti-SARS-CoV-2 S1 IgG, respectively (Table 1).

Among this subpopulation, the vaccine efficacy (VE) in preventing PCR-positive symptomatic COVID-19 was 40.5% (19.8-55.8) in C1; and 65.0% (36.0-80.8), in C2. The VE among the seronegative participants was 54.7% (95% CI: 35.0-68.5), and 64.1% (34.0-80.5) in C, and C2, respectively.

For severe COVID-19 cases, VE could not be estimated due to the small number of events.

© 2023 Mostafavi E et al. *JAMA Network Open.*

eTable 5. Adverse Events (AEs) from Day 0 to day 28

	Vaccine group		Placebo group		P-value
	Events n	Subjects n (%)	Events n	Subjects n (%)	
**Solicited AEs**					
Dose 1	16431	9812 (51.6)	2560	1620 (34.1)	<0.001
Dose 2	13623	8656 (47.8)	2104	1382 (30.6)	<0.001
Dose 3	2465	1493 (35.8)	296	214 (20.4)	<0.001
**Local AEs**					
Dose 1	7877	7269 (38.2)	759	737 (15.5)	<0.001
Dose 2	7013	6470 (35.7)	666	649 (14.4)	<0.001
Dose 3	1376	1042 (25.0)	101	98 (9.3)	<0.001
**Systemic AEs**					
Dose 1	8554	5585 (29.3)	1801	1211 (25.5)	<0.001
Dose 2	6610	4427 (24.4)	1438	977 (21.7)	<0.001
Dose 3	1089	739 (17.7)	195	148 (14.1)	0.005
**AEs within 30 minutes**					
Dose 1	116	109 (0.6)	20	20 (0.4)	0.199
Dose 2	51	39 (0.2)	12	10 (0.2)	0.936
Dose 3	-	-	-	-	-
**Unsolicited in 0-2 days**					
Dose 1	2106	1696 (8.9)	557	458 (9.6)	0.121
Dose 2	1659	1324 (7.3)	421	334 (7.4)	0.823
Dose 3	395	330 (7.9)	73	55 (5.2)	0.003
**AEs in >2 days**					
Dose 1	731	497 (2.6)	183	120 (2.5)	0.734
Dose 2	704	454 (2.5)	201	126 (2.8)	0.274
Dose 3	16	15 (0.4)	1	1 (0.1)	0.221

© 2023 Mostafavi E et al. *JAMA Network Open.*

eTable 6. Local and systemic adverse events after injection of the first, second and third doses

Systemic adverse events	Vaccine groups	Placebo groups	Risk ratio (95% CI)	P value
**Any systemic adverse event**				
Dose 1	5585/19030 (29.35%)	1211/4754 (25.47%)	1·15 (1·09–1·22)	p<0·001
Dose 2	4427/18115 (24.44%)	977/4510 (21.66%)	1·13 (1·06–1·20)	0·003
Dose 3	739/4165 (17.74%)	148/1048 (14.12%)	1·26 (1·07–1·48)	0·095
Fatigue				
Dose 1	2380/19030 (12.51%)	492/4754 (10.35%)	1.21 (1.10-1.32)	0·001
Dose 2	1971/18115 (10.88%)	411/4510 (9.11%)	1.19 (1.08-1.32)	0·014
Dose 3	299/4165 (7.18%)	62/1048 (5.92%)	1.21 (0.93-1.58)	1·000
Headache				
Dose 1	2028/19030 (10.66%)	501/4754 (10.54%)	1.01 (0.92-1.11)	1·000
Dose 2	1488/18115 (8.21%)	349/4510 (7.74%)	1.06 (0.95-1.19)	1·000
Dose 3	227/4165 (5.45%)	51/1048 (4.87%)	1.12 (0.83-1.51)	1·000
Fever				
Dose 1	1244/19030 (6.54%)	237/4754 (4.99%)	1.31 (1.15-1.50)	0·002
Dose 2	819/18115 (4.52%)	177/4510 (3.92%)	1.15 (0.98-1.35)	1·000
Dose 3	151/4165 (3.63%)	23/1048 (2.19%)	1.65 (1.07-2.55)	0·318
Myalgia or asthenia				
Dose 1	1116/19030 (5.86%)	226/4754 (4.75%)	1.23 (1.07-1.42)	0·063
Dose 2	901/18115 (4.97%)	170/4510 (3.77%)	1.32 (1.12-1.55)	0·016
Dose 3	185/4165 (4.44%)	23/1048 (2.19%)	2.02 (1.32-3.11)	0·021
Pain in the extremity				
Dose 1	675/19030 (3.55%)	128/4754 (2.69%)	1.32 (1.09-1.59)	0·070
Dose 2	641/18115 (3.54%)	124/4510 (2.75%)	1.29 (1.06-1.56)	0·148
Dose 3	105/4165 (2.52%)	11/1048 (1.05%)	2.40 (1.30-4.45)	0·074
Chills				
Dose 1	374/19030 (1.97%)	62/4754 (1.30%)	1.51 (1.15-1.97)	0·052
Dose 2	197/18115 (1.09%)	47/4510 (1.04%)	1.04 (0.76-1.43)	1·000

© 2023 Mostafavi E et al. *JAMA Network Open.*

Systemic adverse events	Vaccine groups	Placebo groups	Risk ratio (95% CI)	P value
Dose 3	20/4165 (0.48%)	4/1048 (0.38%)	1.26 (0.43-3.67)	1·000
Diarrhoea				
Dose 1	328/19030 (1.72%)	71/4754 (1.49%)	1.15 (0.89-1.49)	1·000
Dose 2	241/18115 (1.33%)	76/4510 (1.69%)	0.79 (0.61-1.02)	0·976
Dose 3	45/4165 (1.08%)	9/1048 (0.86%)	1.26 (0.62-2.57)	1·000
Nausea or vomiting				
Dose 1	277/19030 (1.46%)	48/4754 (1.01%)	1.44 (1.06-1.96)	0·285
Dose 2	258/18115 (1.42%)	61/4510 (1.35%)	1.05 (0.80-1.39)	1·000
Dose 3	41/4165 (0.98%)	7/1048 (0.67%)	1.47 (0.66-3.28)	<0·001
Rash				
Dose 1	132/19030 (0.69%)	36/4754 (0.76%)	0.92 (0.63-1.32)	1·000
Dose 2	94/18115 (0.52%)	23/4510 (0.51%)	1.02 (0.65-1.60)	0·940
Dose 3	16/4165 (0.38%)	5/1048 (0.48%)	0.81 (0.30-2.19)	1·000
**Local adverse events**				
Any local adverse event				
Dose 1	7269/19030 (38.20%)	737/4754 (15.50%)	2·46 (2·30–2·64)	<0·001
Dose 2	6470/18115 (35.72%)	649/4510 (14.39%)	2·48 (2·31–2·67)	<0·001
Dose 3	1042/4165 (25.02%)	98/1048 (9.35%)	2·68 (2·20–3·25)	<0·001
Pain				
Dose 1	7182/19030 (37.74%)	710/4754 (14.93%)	2.53 (2.36-2.71)	<0·001
Dose 2	6365/18115 (35.14%)	632/4510 (14.01%)	2.51 (2.33-2.70)	<0·001
Dose 3	975/4165 (23.41%)	94/1048 (8.97%)	2.61 (2.14-3.19)	<0·001
Warmth				
Dose 1	211/19030 (1.11%)	23/4754 (0.48%)	2.29 (1.49-3.52)	0·003
Dose 2	150/18115 (0.83%)	10/4510 (0.22%)	3.73 (1.97-7.08)	<0·001
Dose 3	45/4165 (1.08%)	1/1048 (0.10%)	11.32 (1.56-82.05)	0·053
Inflammation				
Dose 1	206/19030 (1.08%)	12/4754 (0.25%)	4.29 (2.40-7.67)	0·001
Dose 2	222/18115 (1.23%)	11/4510 (0.24%)	5.02 (2.75-9.20)	0·001

© 2023 Mostafavi E et al. *JAMA Network Open.*

Systemic adverse events	Vaccine groups	Placebo groups	Risk ratio (95% CI)	P value
Dose 3	171/4165 (4.11%)	2/1048 (0.19%)	21.51 (5.35-86.58)	0·001
Redness				
Dose 1	193/19030 (1.01%)	12/4754 (0.25%)	4.02 (2.24-7.19)	<0·001
Dose 2	203/18115 (1.12%)	10/4510 (0.22%)	5.05 (2.68-9.53)	<0·001
Dose 3	128/4165 (3.07%)	3/1048 (0.29%)	10.74 (3.42-33.66)	<0·001
Induration				
Dose 1	85/19030 (0.45%)	2/4754 (0.04%)	10.62 (2.61-43.13)	0·001
Dose 2	73/18115 (0.40%)	3/4510 (0.07%)	6.06 (1.91-19.21)	0·013
Dose 3	57/4165 (1.37%)	1/1048 (0.10%)	14.34 (1.99-103.46)	0·012

Data are n/N% or risk ratio (95% CI). The safety analysis was done in the per-protocol population, with data from the vaccine groups and the placebo groups in both cohorts combined until the time that participants in cohort 2 alone received dose 3. The risk ratio was the risk in vaccine groups divided by the risk in placebo groups and the 95% CI was calculated as follows: $\exp\{\ln(\widehat{RR}) \pm z_{1-\alpha/2}\sqrt{\frac{(n_1-x_1)/x_1}{n_1}+\frac{(n_2-x_2)/x_2}{n_2}}\}$ where RR is the risk ratio, n_1 is the number of people in the vaccine group, n_2 is the number of people in the placebo group, x_1 is the number of people who had an adverse event in the vaccine group, and x_2 is the number who had an adverse event in the placebo group. Significance values were adjusted for multiple comparisons using the Holm–Bonferroni procedure: first, all p values were sorted from smallest to largest and then second, the $min\{1, (m-i+1) \times p\ value\}$ was considered as the corrected p-value for each, in which m indicates the number of comparisons and i shows the i-th rank.

© 2023 Mostafavi E et al. *JAMA Network Open.*

eTable 7. Serious and medically-attended adverse events (SAEs) from Day 0 up to Day 120

	C1		C2		Total N=23959 n (%)
**Serious and medically-attended adverse events**	**Vaccine group N=14375 n (%)**	**Placebo group N=3597 n (%)**	**Vaccine group N=4790 n (%)**	**Placebo group N=1197 n (%)**	
**Total SAEs**	46 (0.3)	10 (0.3)	9 (0.2)	5 (0.4)	70 (0.3)
Medically-attended	26 (0.2)	5 (0.1)	5 (0.1)	2 (0.2)	38 (0.2)
Serious	16 (0.1)	2 (<0.1)	3 (<0.1)	2 (0.2)	23 (<0.1)
Fatal	4 (<0.1)	3 (<0.1)	1 (<0.1)	1 (<0.1)	9 (<0.1)
**Related to vaccine**	1 (<0.1)	1 (<0.1)	3 (<0.1)	0 (0.0)	5 (<0.1)
**Leading to the discontinuation from the study vaccine**	18 (0.1)	0 (<0.1)	3 (<0.1)	1 (<0.1)	22 (<0.1)
**Leading to the discontinuation from study**	4 (<0.1)	2 (<0.1)	2 (<0.1)	1 (<0.1)	9 (<0.1)

The association of events with injections was categorized according to the causality assessment protocol for AEs[12]

© 2023 Mostafavi E et al. *JAMA Network Open.*

eTable 8. Serious and medically-attended adverse events by MedDRA system organ class during the study, in randomized participants who received at least one dose

Adverse events list according to MedDRA system*	C1		C2	
	Vaccine group N=14375 n (%)	Placebo group N=3597 n (%)	Vaccine group N=14375 n (%)	Placebo group N=3597 n (%)
**Eye disorders**	6 (<0.1)	0 (0.0)	1 (<0.1)	1 (<0.1)
Blurred vision	3	0	1	1
Eye pain	2	0	0	0
Eye floaters	1	0	0	0
**Immune system disorders**	5 (<0.1)	0 (0.0)	0 (0.0)	0 (0.0)
Hypersensitivity reaction	2	0	0	0
Angioedemas	1	0	0	0
Urticaria	2	0	0	0
**Reproductive system and breast disorders**	3 (<0.1)	1 (<0.1)	0 (0.0)	1 (<0.1)
Menometrorrhagia	2	0	0	0
Vaginal bleeding	1	0	0	0
Ovarian cyst	0	0	0	1
**Nervous system disorders**	3 (<0.1)	2 (<0.1)	0 (0.0)	1 (<0.1)
Headache	2	1	0	0
Bell's palsy	1	0	0	1
Paraesthesia lower limb	0	1	0	0
**Blood and lymphatic system disorders**	1 (<0.1)	0 (0.0)	0 (0.0)	0 (0.0)
Lymphadenopathy	1	0	0	0
**General disorders and administration site conditions**	7 (<0.1)	3 (<0.1)	4 (<0.1)	1 (<0.1)
Injection site reaction (erythema, redness and oedema)	0	0	2	0
Unconsciousness	0	1	1	0
Face oedema	2	0	0	0
Non-cardiac chest pain	1	0	0	0
Sudden cardiac death	1	0	0	0
Cardio-respiratory arrest	0	0	0	1
Brain death (due to ventricular arrhythmias and cardiac arrest)	0	0	1	0
Unknown cause of death	1	0	0	0
Accidental death	1	0	0	0
COVID-19 death	1	2	0	0
**Musculoskeletal and connective tissue disorders**	0 (0.0)	0 (0.0)	1 (<0.1)	0 (0.0)

© 2023 Mostafavi E et al. *JAMA Network Open.*

Neck and shoulder pain, with numbness in fingers	0	0	1	0
**Skin and subcutaneous tissue**	3 (<0.1)	1 (<0.1)	0 (0.0)	0 (0.0)
Generalized maculopapular rash	1	1	0	0
Generalized itching	1	0	0	0
Blistering of hand	1	0	0	0
**Gastrointestinal disorders**	2 (<0.1)	1 (<0.1)	1 (<0.1)	0 (0.0)
Abdominal pain and fecal impaction (causing obstruction)	0	0	1	0
Vomiting	1	1	0	0
Haematochezia and [illegible]	1	0	0	0
**Obstetric and gynaecological therapeutic procedures**	2 (<0.1)	0 (0.0)	0 (0.0)	0 (0.0)
Induced abortions	2	0	0	0
**Pregnancy, puerperium and perinatal conditions**	3 (<0.1)	1 (<0.1)	1 (<0.1)	0 (0.0)
Spontaneous abortion	3	0	1	0
Ectopic pregnancy	0	1	0	0
**Renal and urinary disorders**	7 (<0.1)	0 (0.0)	0 (0.0)	1 (<0.1)
Haematuria	5	0	0	1
Nephritis	1	0	0	0
Dysuria	1	0	0	0
**Respiratory, thoracic, and mediastinal disorders**	2 (<0.1)	1 (<0.1)	0 (0.0)	0 (0.0)
Chest pain and Dyspnoea	1	0	0	0
Bronchopneumonia	0	1	0	0
Dyspnoea	1	0	0	0
**Cardiac disorders**	3 (<0.1)	0 (0.0)	1 (<0.1)	1 (<0.1)
Myocardial infarction	1	0	1	0
Chest pain – cardiac	1	0	0	0
Arrhythmia and Palpitation	1	0	0	0
Palpitation	0	0	0	1
**Vascular Disorders**	2 (<0.1)	0 (0.0)	0 (0.0)	1 (<0.1)
Ischemic heart disease (the leading cause of death)	1	0	0	0
Dizziness	1	0	0	1
**Injury, poisoning and procedural complications**	2 (<0.1)	1 (<0.1)	0 (0.0)	0 (0.0)
Knee injury	1	0	0	0
Traumatic intracranial [illegible]	0	1	0	0
Traumatic hand fracture	1	0	0	0
**Neoplasms benign, malignant [illegible]**	2 (<0.1)	0 (0.0)	0 (0.0)	0 (0.0)
Cholangiocarcinoma	1	0	0	0
Laryngeal cancer	1	0	0	0

Note: *Some participants had more than one adverse events symptom; MedDRA: Medical Dictionary for Regulatory Activities Terminology

© 2023 Mostafavi E et al. *JAMA Network Open.*

eTable 9. The Number Needed to Vaccinate in Cohort 1 and Cohort 2

**Cohort**	$NNV*$	95% CI
Cohort 1: 14 days after the second dose	32.3	24.4, 47.6
Cohort 2: 14 days after the third dose	29.4	20.0, 55.6

NNV: Number needed to vaccinate. NNV is estimated using the cox regression model

© 2023 Mostafavi E et al. *JAMA Network Open.*

eTable 10. Reason for unmet inclusion criteria in screened participants

**Reason**	**Number of participants**
Uncontrolled underlying diseases (type 2 diabetes mellitus, chronic renal disease, chronic liver disease)	40
Immunomodulators/immunosuppressive usage	10
History of psychological disorders	5
Heavy smoker	2
History of allergy	2
History of tetanus toxoid vaccination within 3 months before the current study	1
Other	10

Among 24,126 participants who were screened, 70 participants did not meet the inclusion criteria and so, were not included in the study. In this regard, uncontrolled underlying diseases such as type 2 diabetes, chronic renal disease, respiratory disease, and chronic liver disease were among the most common reasons.

© 2023 Mostafavi E et al. *JAMA Network Open.*

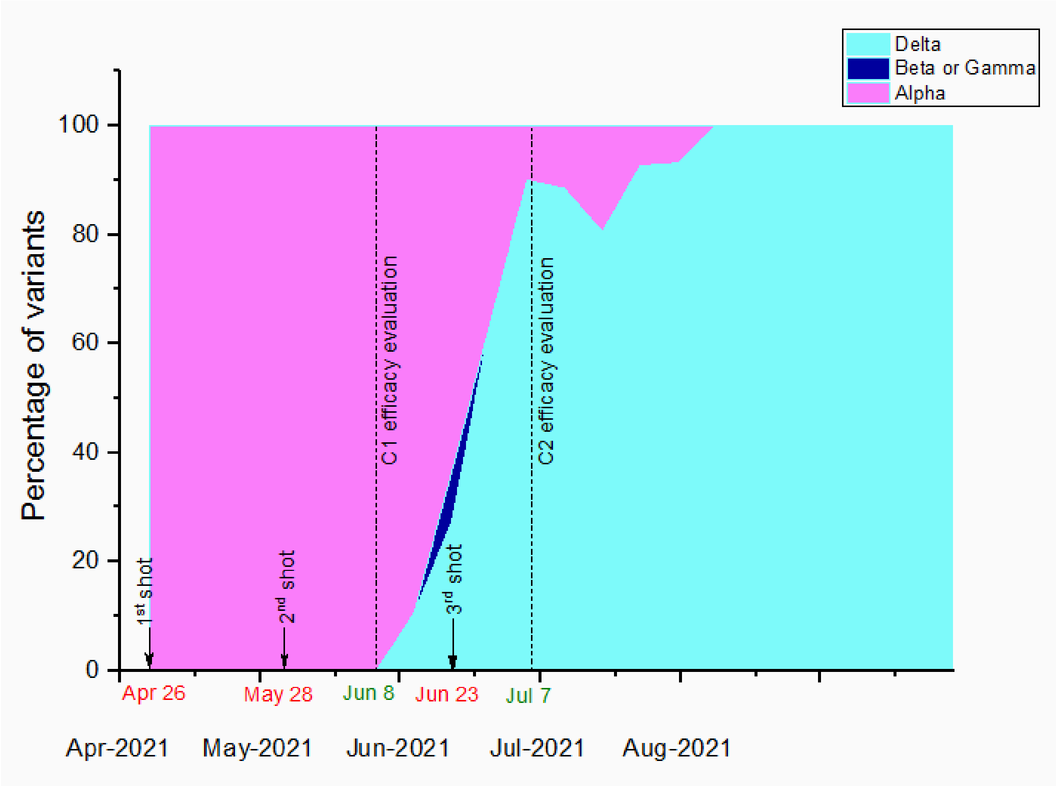

eFigure 4. Percentage of different SARS-CoV-2 variants of concern during the study

Note: 26-April start of the 1st dose, the 25-May start of the second dose, the 23-June start of the third dose. The dashed lines show the start of the C1 efficacy evaluation on 8 June and the start of the C2 efficacy evaluation on 7 July. (n=419 sequenced samples).

© 2023 Mostafavi E et al. *JAMA Network Open.*

eReferences

1. Pérez-Rodríguez S, de la Caridad Rodríguez-González M, Ochoa-Azze R, et al. A randomized, double-blind phase I clinical trial of two recombinant dimeric RBD COVID-19 vaccine candidates: Safety, reactogenicity and immunogenicity. *Vaccine* 2022; **40**(13): 2068-75.
2. Eugenia-Toledo-Romaní M, Verdecia-Sánchez L, Rodríguez-González M, et al. Safety and immunogenicity of anti-SARS CoV-2 vaccine SOBERANA 02 in homologous or heterologous scheme: Open label phase I and phase IIa clinical trials. *Vaccine* 2022; **40**(31): 4220-30.
3. Toledo-Romani ME, García-Carmenate M, Verdecia-Sánchez L, et al. Safety and immunogenicity of anti-SARS-CoV-2 heterologous scheme with SOBERANA 02 and SOBERANA Plus vaccines: Phase IIb clinical trial in adults. *Med (N Y)* 2022; **3**(11): 760-73.e5.
4. Ochoa-Azze R, Chang-Monteagudo A, Climent-Ruiz Y, et al. Safety and immunogenicity of the FINLAY-FR-1A vaccine in COVID-19 convalescent participants: an open-label phase 2a and double-blind, randomised, placebo-controlled, phase 2b, seamless, clinical trial. *The Lancet Respiratory Medicine* 2022.
5. Statistical Centre of Iran. Findings of national population and household census. 2020. https://www.amar.org.ir/english/Population-and-Housing-Censuses (accessed 24 December 2021).
6. Worldometers. Iran COVID - Coronavirus Statistics - Worldometer. 2021. https://www.worldometers.info/coronavirus/country/iran/ (accessed December 23 2021).
7. Ella R, Reddy S, Blackwelder W, et al. Efficacy, safety, and lot-to-lot immunogenicity of an inactivated SARS-CoV-2 vaccine (BBV152): interim results of a randomised, double-blind, controlled, phase 3 trial. *The Lancet* 2021; **398**(10317): 2173-84.
8. Tanriover MD, Doğanay HL, Akova M, et al. Efficacy and safety of an inactivated whole-virion SARS-CoV-2 vaccine (CoronaVac): interim results of a double-blind, randomised, placebo-controlled, phase 3 trial in Turkey. *The Lancet* 2021; **398**(10296): 213-22.
9. Hernán MA, Robins JM. Causal inference. CRC Boca Raton, FL;; 2010.
10. Hernán MA. The hazards of hazard ratios. *Epidemiology (Cambridge, Mass)* 2010; **21**(1): 13.
11. Dagan N, Barda N, Kepten E, et al. BNT162b2 mRNA Covid-19 vaccine in a nationwide mass vaccination setting. *New England Journal of Medicine* 2021.
12. World Health O. Causality assessment of an adverse event following immunization (AEFI): user manual for the revised WHO classification. 2nd , 2019 update ed. Geneva: World Health Organization; 2019.

© 2023 Mostafavi E et al. *JAMA Network Open.*
