# Supplementary material for: Efficacy and Safety of a Protein-Based SARS-CoV-2 Vaccine: A Randomized Clinical Trial
Source: JAMA Netw Open. 2023 May 3;6(5):e2310302. doi: 10.1001/jamanetworkopen.2023.10302 (PMC10157429; doi:10.1001/jamanetworkopen.2023.10302)
Supplement: Supplement 3. — Nonauthor Collaborators. The Soberana Study Group [file jamanetwopen-e2310302-s003.pdf]

Supplemental Online Content: Nonauthor Collaborators
*First name, last name, and suffix (if applicable) are required and will appear in PubMed.

*Group Name(s): The SOBERANA Study Group									
***First Name and Middle Initial(s)**	***Last Name**	***Suffix (eg, Jr, III)**	Academic Degrees	Institution	Location (city, state/province, country)	Role or Contribution, eg, chair, principal investigator	Group (if more than 1 Group listed in the byline) and/or Subgroup (eg, Steering Committee)		
Behnaz	Soleimani Tappeh Sari			Babol University of Medical Sciences	Mazandaran, Babol, Iran	Executive manager			
Soheil	Ebrahimpour			Babol University of Medical Sciences	Mazandaran, Babol, Iran	Vaccination site manager			
Rostam	Heydar Tabar			Babol University of Medical Sciences	Mazandaran, Babol, Iran	Vaccination site manager			
Roghayye	Vaseghi			Babol University of Medical Sciences	Mazandaran, Babol, Iran	Research physician			
Bahram	Mirzaei			Babol University of Medical Sciences	Mazandaran, Babol, Iran	Research physician			
Mehdi	Safarpour			Babol University of Medical Sciences	Mazandaran, Babol, Iran	System Administrator			
Farzin	Sadeghi			Babol University of Medical Sciences	Mazandaran, Babol, Iran	Laboratory executive			
Mahmoud	Hosseinpoor			Hormozgan University of Medical Sci	Hormozgan, Bandar Abbas	Senior executive manager			
Tayebeh	Raznahan			Hormozgan University of Medical Sci	Hormozgan, Bandar Abbas	Executive manager			
Leila	Haddadi			Hormozgan University of Medical Sci	Hormozgan, Bandar Abbas	Vaccination site manager			
Arezoo	Mobarak Abadi			Hormozgan University of Medical Sci	Hormozgan, Bandar Abbas	Vaccination site manager			
Elham	Khalili			Hormozgan University of Medical Sci	Hormozgan, Bandar Abbas	Research physician			
Mehrsa	Manaei			Hormozgan University of Medical Sci	Hormozgan, Bandar Abbas	Research physician			
Hassan	Morshedi			Hormozgan University of Medical Sci	Hormozgan, Bandar Abbas	System Administrator			
Hamed	Gooklani			Hormozgan University of Medical Sci	Hormozgan, Bandar Abbas	Laboratory executive			
Manouchehr	Karami			Hamadan University of Medical Scier	Hamadan, Iran	Senior executive manager			
Mohammad	Khazaei			Hamadan University of Medical Scier	Hamadan, Iran	Senior executive manager			
Seyyed Jalaleddin	Bathaei			Hamadan University of Medical Scier	Hamadan, Iran	Executive manager			
Ali	Ataei			Hamadan University of Medical Scier	Hamadan, Iran	Executive manager			
Ali	Zahiri			Hamadan University of Medical Scier	Hamadan, Iran	Vaccination site manager			
Zahra	Eskandari			Hamadan University of Medical Scier	Hamadan, Iran	Vaccination site manager			
Firouzeh	Karbalaei Zadeh			Hamadan University of Medical Scier	Hamadan, Iran	Research physician			
Elham	Abdoli			Hamadan University of Medical Scier	Hamadan, Iran	Research Physician			
Marzieh	Arjmandian			Hamadan University of Medical Scier	Hamadan, Iran	Research Physician			
Maryam	Zamanian			Hamadan University of Medical Scier	Hamadan, Iran	Research Physician			
Razieh	Sadat Mirmoeini			Hamadan University of Medical Scier	Hamadan, Iran	Research Physician			
Seyyede Zeinab	Hashemi			Hamadan University of Medical Scier	Hamadan, Iran	System Administrator			
Farid	Azizi Jalilian			Hamadan University of Medical Scier	Hamadan, Iran	Laboratory executive			
Kamal	Heidari			Deputy of Health, Isfahan University	Isfahan, Iran	Senior executive manager			
Mehdi	Farsi			CDC, Isfahan University of Medical Sc	Isfahan, Iran	Executive manager			
Mahnaz	Mostajeran			Department of Demography, Health	Isfahan, Iran	Vaccination site manager			
Marjan	Mashkooti			Isfahan University of Medical Science	Isfahan, Iran	Research physician			
Somayye	Davashi			Isfahan University of Medical Science	Isfahan, Iran	Research physician			
Nastaran	Kian Poor			Isfahan University of Medical Science	Isfahan, Iran	Research physician			

Supplemental Online Content: Nonauthor Collaborators
*First name, last name, and suffix (if applicable) are required and will appear in PubMed.

*First Name and Middle Initial(s)	*Last Name	*Suffix (eg, Jr, III)	Academic Degrees	Institution	Location (city, state/province, country)	Role or Contribution, eg, chair, principal investigator	Group (if more than 1 Group listed in the byline) and/or Subgroup (eg, Steering Committee)		
Asefeh	Haddadpour			Isfahan University of Medical Science	Isfahan, Iran	System Administrator			
Fariba	Mazrooei			Isfahan University of Medical Science	Isfahan, Iran	Laboratory executive			
Hossein	Safizadeh			Kerman University of Medical Scienc	Kerman, Iran	Senior executive manager			
Mohammadreza	Naghavi			Kerman University of Medical Scienc	Kerman, Iran	Executive manager			
Saeed	Sohbati			Kerman University of Medical Scienc	Kerman, Iran	Research physician			
Nadia	Mohit Kermani			Kerman University of Medical Scienc	Kerman, Iran	Research physician			
Nasim	Nasiri Moghaddam			Kerman University of Medical Scienc	Kerman, Iran	System Administrator			
Shahriar	Dabiri			Kerman University of Medical Scienc	Kerman, Iran	Laboratory executive			
Ghasem	Oveis			Mazandaran University of Medical Sc	Mazandaran, Sari, Iran	Senior executive manager			
Mohammadreza	Parsaei			Mazandaran University of Medical Sc	Mazandaran, Sari, Iran	Executive manager & vaccination site manager			
Maghsood	Khalizadeh			Mazandaran University of Medical Sc	Mazandaran, Sari, Iran	Research physician			
Abbas	Arjmand			Mazandaran University of Medical Sc	Mazandaran, Sari, Iran	Research physician			
Fatemeh	Yazdi Zadeh			Mazandaran University of Medical Sc	Mazandaran, Sari, Iran	Research physician			
Hadi	Dehbashi araghdari			Mazandaran University of Medical Sc	Mazandaran, Sari, Iran	System Administrator			
Zainab	Bandalizadeh			Mazandaran University of Medical Sc	Mazandaran, Sari, Iran	Laboratory executive			
Mohammadreza	Dehghani			Yazd University of Medical Sciences	Yazd, Iran	Senior executive manager			
Mehrdad	Mansouri			Yazd University of Medical Sciences	Yazd, Iran	Vaccination site manager and research physician			
Masoud	Sharifi			Yazd University of Medical Sciences	Yazd, Iran	Executive manager			
Hossein	Shojaei Far			Yazd University of Medical Sciences	Yazd, Iran	Executive manager			
Javad	Soheili			Yazd University of Medical Sciences	Yazd, Iran	Executive manager			
Sina	Owlia			Yazd University of Medical Sciences	Yazd, Iran	Research physician			
Masoumeh	Mazidi			Yazd University of Medical Sciences	Yazd, Iran	Research physician			
Mohammad	Sharif Yazdi			Yazd University of Medical Sciences	Yazd, Iran	Scientific manager			
Mahdieh	Bahri			Yazd University of Medical Sciences	Yazd, Iran	Research physician			
Razieh Sadat	Hashemi			Yazd University of Medical Sciences	Yazd, Iran	Research physician			
Alireza	Talebi			Yazd University of Medical Sciences	Yazd, Iran	Laboratory executive			
Saeideh	Anvari			Yazd University of Medical Sciences	Yazd, Iran	System Administrator			
Ahmad	Jafari			Yazd University of Medical Sciences	Yazd, Iran	Vaccination site manager			
Mohammadreza	Saeini			Center for Diseases Control, Zanjan U	Zanjan, Iran	Senior executive manager			
Fariba	Najafi			Zanjan University of Medical Science	Zanjan, Iran	Vaccination site manager			
Davoud	Rahmani			Zanjan University of Medical Science	Zanjan, Iran	Research physician			
Ali	Seidy			Zanjan University of Medical Science	Zanjan, Iran	Research physician			
Mahshid	Hajikhani			Zanjan University of Medical Science	Zanjan, Iran	System Administrator			
Hossein	Dinmohammadi			Zanjan University of Medical Science	Zanjan, Iran	Laboratory executive			
Amir	Javadi			Department of Community Medicine	Ghazvin, Iran	A member of the Monitoring and Audit team			
Seyed Ebrahim	Eskandari			Center for Research and Training in S	Tehran, Iran	A member of the Monitoring and Audit team			

Supplemental Online Content: Nonauthor Collaborators

*First name, last name, and suffix (if applicable) are required and will appear in PubMed.

***First Name and Middle Initial(s)**	***Last Name**	***Suffix (eg, Jr, III)**	Academic Degrees	Institution	Location (city, state/province, country)	Role or Contribution, eg, chair, principal investigator	Group (if more than 1 Group listed in the byline) and/or Subgroup (eg, Steering Committee)		
Farhad	Handjani			Department of Dermatology, School	Fars, Shiraz , Iran	A member of the Monitoring and Audit team			
Maryam	Chegeni			Molecular and Medicine Research Ce	Markazi, Khomein, Iran	A member of the team of technical consultants			
Katayoun	Seif Farahi			Center for Communicable Diseases C	Tehran, Iran	A member of the team of technical consultants			
Payman	Hemmati			Center for Communicable Diseases C	Tehran, Iran	A member of the team of technical consultants			
Mahsa	Tavakoli Rad			National Reference Laboratory for C	Tehran, Iran	A member of the COVID-19 National Reference Laboratory team			
Zahra	Fereidooni			National Reference Laboratory for C	Tehran, Iran	A member of the COVID-19 National Reference Laboratory team			
Sahar	Khakifirouz			National Reference Laboratory for C	Tehran, Iran	A member of the COVID-19 National Reference Laboratory team			
Tahereh	Mohammadi			National Reference Laboratory for C	Tehran, Iran	A member of the COVID-19 National Reference Laboratory team			
Mehdi	Fazlalipour			National Reference Laboratory for C	Tehran, Iran	A member of the COVID-19 National Reference Laboratory team			
Ali	Maleki			National Reference Laboratory for C	Tehran, Iran	A member of the COVID-19 National Reference Laboratory team			
Amir Hesam	Nemati			National Reference Laboratory for C	Tehran, Iran	A member of the COVID-19 National Reference Laboratory team			
Ahmad	Ghasemi			National Reference Laboratory for C	Tehran, Iran	A member of the COVID-19 National Reference Laboratory team			
Sanam	Azad Manjiri			National Reference Laboratory for C	Tehran, Iran	A member of the COVID-19 National Reference Laboratory team			
Zahra	Ahmadi			National Reference Laboratory for C	Tehran, Iran	A member of the COVID-19 National Reference Laboratory team			
Parastoo	Yekta Sanati			National Reference Laboratory for C	Tehran, Iran	A member of the COVID-19 National Reference Laboratory team			
Setareh	Kashanian			National Reference Laboratory for C	Tehran, Iran	A member of the COVID-19 National Reference Laboratory team			
Mohammad Mehdi	Mortazavipoor			National Reference Laboratory for C	Tehran, Iran	A member of the COVID-19 National Reference Laboratory team			
Laya	Farhan Asadi			National Reference Laboratory for C	Tehran, Iran	A member of the COVID-19 National Reference Laboratory team			
Zahra	Hosseini			National Reference Laboratory for C	Tehran, Iran	A member of the COVID-19 National Reference Laboratory team			
Farideh	Niknam Oskouei			National Reference Laboratory for C	Tehran, Iran	A member of the COVID-19 National Reference Laboratory team			
Sepideh	Gerdooie			National Reference Laboratory for C	Tehran, Iran	A member of the COVID-19 National Reference Laboratory team			
Marzyie	Sajadi			National Reference Laboratory for C	Tehran, Iran	A member of the COVID-19 National Reference Laboratory team			
Maryam	Rostamtabar			National Reference Laboratory for C	Tehran, Iran	A member of the COVID-19 National Reference Laboratory team			
Talieh	Sabouni			Production and Research Complex, P	Tehran, Iran	A member of the Manufacture, Packaging and Labeling team			
Alireza	Rahimi			Production and Research Complex, P	Tehran, Iran	A member of the Manufacture, Packaging and Labeling team			
Alireza	Kavianpour			Production and Research Complex, P	Tehran, Iran	A member of the Manufacture, Packaging and Labeling team			
Seyed Mohsen	Zahraei			Center for Communicable Disease Co	Tehran, Iran	A member of the Adverse Event Evaluation Committee			
Katayoun	Tayeri			National HIV/AIDS Care & Treatment	Tehran, Iran	A member of the Adverse Event Evaluation Committee			
Ali	Asadollahi Amin			Iranian Research Center for HIV/AIDS	Tehran, Iran	A member of the Adverse Event Evaluation Committee			
Hamid Reza	Tohidinik			HIV/STI Surveillance Research Center	Kerman, Iran	A member of the Data Analysis Committee			
Azam	Rastegari			Modeling in Health Research Center,	Kerman, Iran	A member of the Data Analysis Committee			
Soheil	Mehmandoost			HIV/STI Surveillance Research Center	Kerman, Iran	A member of the Data Analysis Committee			
