# Supplementary material for: Efficacy and Safety of a Protein-Based SARS-CoV-2 Vaccine: A Randomized Clinical Trial
Source: JAMA Netw Open. 2023 May 3;6(5):e2310302. doi: 10.1001/jamanetworkopen.2023.10302 (PMC10157429; doi:10.1001/jamanetworkopen.2023.10302)
Supplement: Supplement 4. — Data Sharing Statement [file jamanetwopen-e2310302-s004.pdf]

Data Sharing Statement

Mostafavi. Efficacy and Safety of a Protein-Based SARS-CoV-2 Vaccine. *JAMA Netw Open*. Published May 03, 2023. doi:10.1001/jamanetworkopen.2023.10302

Data
Data available: Yes
Data types: Deidentified participant data
How to access data: Deidentified participant data are available with permission of Pasteur Institute of Iran from the corresponding author (Dr Alireza Biglari, email: Biglari63@hotmail.com) upon reasonable request.
When available: With publication

Supporting Documents
Document types: None

Additional Information
Who can access the data: Researchers whose proposed use of the data has been approved
Types of analyses: For any purpose
Mechanisms of data availability: With investigator support and after approval of a proposal
